# The relationship between latex metabolism gene expression with rubber yield and related traits in *Hevea brasiliensis*

**DOI:** 10.1186/s12864-018-5242-4

**Published:** 2018-12-10

**Authors:** Chuntai Wu, Li Lan, Yu Li, Zhiyi Nie, Rizhong Zeng

**Affiliations:** 1 Ministry of Agriculture Key Laboratory of Biology and Genetic Resources of Rubber Tree, Rubber Research Institute, Chinese Academy of Tropical Agricultural Sciences (CATAS), Danzhou, Hainan 571737 People’s Republic of China; 2 College of Agriculture, Hainan University, Haikou, 570228 China

**Keywords:** *Hevea brasiliensis*, Latex metabolism-related genes, Rubber yield, Yield characteristics, Real-time quantitative RT-PCR

## Abstract

**Background:**

Expression patterns of many laticifer-specific gens are closely correlative with rubber yield of *Hevea brasiliensis* (para rubber tree). To unveil the mechanisms underlying the rubber yield, transcript levels of nine major latex metabolism-related genes, i.e., HMG-CoA synthase (HMGS), HMG-CoA reductase (HMGR), diphosphomevalonate decarboxylase (PMD), farnesyl diphosphate synthase (FPS), *cis*-prenyltransferase (CPT), rubber elongation factor (REF), small rubber particle protein (SRPP), dihydroxyacid dehydratase (DHAD) and actin depolymerizing factor (ADF), were dertermined, and the relationship between rubber yield with their expression levels was analysed.

**Results:**

Except *HbHMGR1*, *HbPMD* and *HbDHAD*, most of these genes were predominantly expressed in latex, and bark tapping markedly elevated the transcript abundance of the analyzed genes, with the 7th tapping producing the greatest expression levels. Both ethephon (ETH) and methyl jasmonate (MeJA) stimulation greatly induced the expression levels of the examined genes, at least at one time point, except *HbDHAD*, which was unresponsive to MeJA. The genes’ expression levels, as well as the rubber yields and two yield characteristics differed significantly among the different genotypes examined. Additionally, the latex and dry rubber yields increased gradually but the dry rubber content did not. Rubber yields and/or yield characteristics were significantly positively correlated with *HbCPT*, *HbFPS*, *HbHMGS*, *HbHMGR1* and *HbDHAD* expression levels, negatively correlated with that of *HbREF*, but not significantly correlated with *HbPMD*, *HbSRPP* and *HbADF* expression levels. In addition, during rubber production, significantly positive correlations existed between the expression level of *HbPMD* and the levels of *HbREF* and *HbHMGR1*, between *HbSRPP* and the levels of *HbHMGS* and *HbHMGR1*, and between *HbADF* and *HbFPS*.

**Conclusions:**

The up-regulation of these genes might be related to the latex production of rubber trees under the stress of bark tapping and latex metabolism. The various correlations among the genes implied that there are differences in their synergic interactions. Thus, these nine genes might be related to rubber yield and yield-related traits in *H. brasiliensis*, and this work increases our understanding of their complex functions and how they are expressed in both high-and medium-yield rubber tree varieties and low-yield wild rubber tree germplasm.

**Electronic supplementary material:**

The online version of this article (10.1186/s12864-018-5242-4) contains supplementary material, which is available to authorized users.

## Background

Natural rubber, composed mainly of *cis*-1,4-polyisoprene, is an important raw material of high economic value from which hundreds of industrial and medical products are manufactured [1]. Currently, more than 2500 species of latex-producing plants have been reported [2, 3]. In addition, both guayule (*Parthenium argentatum*) and dandelion (*Taraxacum* sp.) are alternative sources of natural rubber [4]. The perennial tropical cash crop, para rubber tree (*Hevea brasiliensis*), belonging to the *Euphorbiaceae* family, which is widely planted in Southeast Asia, is still prevalent because of its greater productivity than the sum total of all other latex-producing plants, including guayule and dandelion, and because of the outstanding physical properties of its rubber products [5]. *Hevea* latex is the main worldwide resource of commercial natural rubber, accounting for up to 90% of the rubber traded in global markets [2, 6, 7].

Natural rubber belonging to the isoprenoid family of plant natural products is biosynthesized using pyruvate-derived acetyl-CoA as a substrate through the cytosolic mevalonate (MVA) pathway [8]. Many enzymes and proteins found in latex are implicated in this process. The biosynthetic pathway of rubber can be generally divided into three stages. At the initial and later stages, isopentenyl diphosphate (IPP), which is the basic carbon skeleton for universal isoprenoid biosynthesis, and its allylic isomer dimethylallyl diphosphate, which is the starter molecule for subsequent additions of IPP, are synthesized through the MVA pathway from their initial donor, pyruvate metabolite acetyl coenzyme A (acetyl-CoA). The biosynthesis of IPP in the MVA pathway requires a series of six enzymatic reactions. Initially, three molecules of acetyl-CoA are converted to MVA consecutively through acetoacetyl-CoA and 3-hydroxy-3-methylglutaryl (HMG)-CoA. MVA is then sequentially phosphorylated and ATP-dependently decarboxylated to ultimately form IPP [9–11]. In this second step of the MVA pathway involving the synthesis of IPP, acetoacetyl-CoA and acetyl-CoA are aldol-condensed to HMG-CoA by HMG-CoA synthase (HMGS, EC 2.3.3.10), and HMG-CoA is further converted to MVA by HMG-CoA reductase (HMGR, EC 1.1.1.34). In the last step of the conversion of acetyl-CoA to IPP, the latter is produced from diphosphomevalonate by diphosphomevalonate decarboxylase (PMD, EC 4.1.1.33). At the middle stage, IPP is sequentially condensed with dimethylallyl diphosphate to produce geranyl diphosphate (GPP), with GPP to form farnesyl diphosphate (FPP), and with FPP to produce geranylgeranyl diphosphate [12, 13]. T*rans*-FPP is synthesized from GPP by the enzyme FPP synthase (FPS, EC 2.5.1.1) [14]. *cis*-Prenyltransferase (CPT, EC 2.5.1.20) catalyzes the sequential *cis*-1,4-condensation of IPP with *cis*-FPP and its growing chains [8, 15, 16]. At the later stages, in which the allylic primer for the *cis*-1,4-polymerization of isoprene units from IPP is comprised of GDP, FDP and GGDP, rubber hydrocarbons of *cis*-1,4-polyisoprene are synthesized mainly by adding approximately 15,000 IPP molecules to an FPP molecule in a *cis*-1,4-configuration [8, 17]. Rubber elongation factor (REF) is required for different prenyltransferases from broad sources to add a number of *cis*-IPP molecules to rubber chains [18]. In both large and small rubber particles, REF and small rubber particle protein (SRPP) form a dense proteolipidic monomembrane with the lipid monolayer that contributes to the colloidal stability of latex [19].

In addition to the well-documented substrate acetyl-CoA in the MVA pathway, IPP is also synthesized from pyruvate and glyceraldehyde-3-phosphate through the recently discovered methyl-erythritol 4-phosphate (MEP) pathway in the chloroplasts of plants [20]. Furthermore, other sources, such as leucine amino acid metabolism, provide the intermediate 3-hydroxy-3-methylglutaryl coenzyme A for IPP backbone synthesis [21]. Dihydroxyacid dehydratase (DHAD, EC 4.2.1.9) catalyzes a key step in the biosynthetic pathway producing the branched-chain amino acids isoleucine, valine and leucine that exists in plants [22]. This enzyme is stress labile because of its Fe-S cluster [23].

Laticifers in the phloem (inner bark) tissue of rubber trees are highly specialized cells for rubber production and comprise an effective defense system against environmental stress [24, 25]. Currently, latex is commercially removed from laticifer cells by regularly slicing the mature stem bark of rubber trees until the wounded laticifer is blocked, causing the latex drainage to cease [7, 26, 27]. Actin depolymerizing factor (ADF), an important actin-binding protein, plays a role in the plugging process of laticifers [28].

Accordingly, the transcriptional levels of genes encoding these enzymes or proteins in latex are closely related to latex metabolism, including rubber biosynthesis, in *Hevea* trees. The sequences of latex metabolism-related genes have been reported, and expression analyses have been conducted in the latex of a few *H. brasiliensis* varieties [24, 29–33]. However, the simultaneous characterization of these genes in diverse genotypes of rubber trees has not been performed to date. To investigate the unitary molecular events concerned with latex metabolism, we performed expression analyses on nine latex metabolism-related genes in various tissues of newly tapped young ‘CATAS 73397’ rubber trees and in latex from trees under various abiotic stresses, including mechanical wounding (tapping) and ethephon (ETH) and methyl jasmonate (MeJA) treatments. In addition, a comparative expression analysis of the aforementioned genes was conducted using different *Hevea* clones, including high- and medium-yield varieties and low-yield germplasm. A variation analysis for rubber yield and its related traits in high- and medium-yield varieties was conducted, as was a correlation analysis of gene expression levels in high- and medium-yield varieties with rubber yields and yield-related traits. Our results produced valuable insights into the functions of individual genes in latex metabolism.

## Methods

### Planting materials

This study involved 20 *H. brasiliensis* clones from seven cultivars already recommended for cultivation in China (RRIM 600, PR 107, TSF 523, TSF 628, CATAS 73397, CATAS 72059 and CATAS 879), three strains under a trial planting period (TSF 192, CATAS 78426 and CATAS 87662) and ten low-yield wild germplasm (RO/J/6 32/35, MT/IT/13 29/8, MT/C/2 10/49, RO/J/6 32/49, RO/PB/1 2/78, MT/IT/14 30/18, RO/C/9 23/219, RO/A/7 25/198, AC/F/7 38/63 and AC/AB/15 54/980). These clones were cultivated in 2006 at the Danzhou Experimental Farm, Chinese Academy of Tropical Agricultural Sciences (CATAS) in Hainan, P.R. China, in a randomized block design with three replicates. Each independent experimental unit of the different tested cultivars comprised ~ 56 trees (seven lines of eight trees), in an area of approximately 0.12 ha. Each tested wild germplasm consisted of four trees, and the row and plant spacing were 7 m and 3 m, respectively. The trees were opened for tapping in the eighth year after cultivating to a height of 120 cm above the highest point of the bud union. The tapping of all trees for the collection of latex was done every 3 d using a half spiral system (1/2S D/3) without ETH stimulation on panel BO-1. In the event of bad weather (such as rainy days), tapping was delayed until the following day. Tender leaves, male and female flowers, stem bark and latex used for the qRT-PCR experiments were collected from seven-year-old untapped clonal rubber trees of ‘CATAS 73397’. ETH and MeJA treatments before opening the rubber tree for tapping and fresh latex collection and preservation were conducted as previously described [25, 34].

### Total RNA extraction and cDNA synthesis

In the experiment, each sample included three independent biological replicates, and each biological replicate comprised at least 5 individual trees from which latex was collected and pooled as described previously [35]. Briefly, fresh latex was transferred to a vacuum bottle containing liquid nitrogen 30 s after tapping, and then immediately used or stored frozen at − 80 °C. Total RNA was extracted from the fresh latex using the improved sodium dodecyl sulfonate method [36, 37], and further treated with RNase-free DNase I (TaKaRa) to eliminate the residual genomic DNA. To check for DNA contamination, the RNA sample was subsequently used as a template for 18S gene amplification. The cDNA was synthesized from 2 μg of total RNA of latex samples from 20 types of rubber tree clones using a RevertAid™ First-Strand cDNA Synthesis Kit (^#^K1621; Fermentas, Lithuania) containing an optimized mix of oligo(dT) and random primers following the supplier’s instructions. Each cDNA sample was diluted 10-fold with sterilized ddH_2_O, and 1 μl of the 1:10 dilution was employed as template for qRT-PCR.

### Gene expression analysis by RT-qPCR

Based on the corresponding rubber tree EST sequences deposited in NCBI database with the accession numbers listed in Additional file 1: Table S1, specific primer pairs having a Tm range of 56–61 °C and flanking amplicon sizes of 100–200 bp were designed for nine *Hevea* genes (*HbHMGS*, *HbHMGR1*, *HbPMD*, *HbFPS*, *HbCPT*, *HbREF*, *HbSRPP*, *HbADF*, and *HbDHAD*) related to latex metabolism using the Beacon Designer version 8.13 software (Premier Biosoft International, USA) (Additional file 1: Table S1). Real-time PCR was carried out in a 20-μL reaction system independently using 20 serial 10-fold dilutions of single-stranded latex transcripts as templates with primer pairs (0.5 pmol) for each of the nine genes from all of the compared clones. The 18 s RNA gene was selected as an internal control. We performed the quantitative gene expression analysis on a LightCycler 2.0 instrument (Roche, Basel, Switzerland). The optimum PCR procedure conditions were denaturation at 95 °C for 3 min, 39 cycles with denaturation at 94 °C for 10 s, annealing at 58 °C for 15 s and extension at 72 °C for 30 s, followed by a last extension at 72 °C for 10 min. All qRT-PCR reactions were conducted in three biological replicates each with technical triplicates. Based on the relative quantification method described by Pfaffl [38], the transcript levels were calculated by normalization relative to the transcript abundance of the 18S reference gene on a LightCycler 4.05.

### Rubber yield estimation

Every 10 d throughout the year, latex volumes from each experimental unit were determined by applying the methods of individual collection and measurement. Latex dry rubber contents (DRCs) were measured by collecting latex from no less than five individual plants per experimental unit, and the dry matter productivity of the rubber trees was calculated in gram per tree per tapping.

### Data analysis

To determine significant differences, data from the gene expression analysis and rubber production determination were subjected to Duncan’s new multiple comparison test and analyzed using the SAS system 8.0 (SAS Institute Inc., Cary, NC, USA). The mRNA expression levels of nine genes at the three tapping times examined and all of the tested clones, as well as yield and yield-related traits among the tested cultivars, were compared, and pairwise correlations were computed between all tapping times and all tested clones’ gene expression data or between all of the tested cultivars’ gene expression data and corresponding available yield and yield-related trait data.

## Results

### Expression profiles of latex metabolic genes in different tissues of young mature *Hevea* ‘CATAS 73397’ trees

**Fig. 1:**
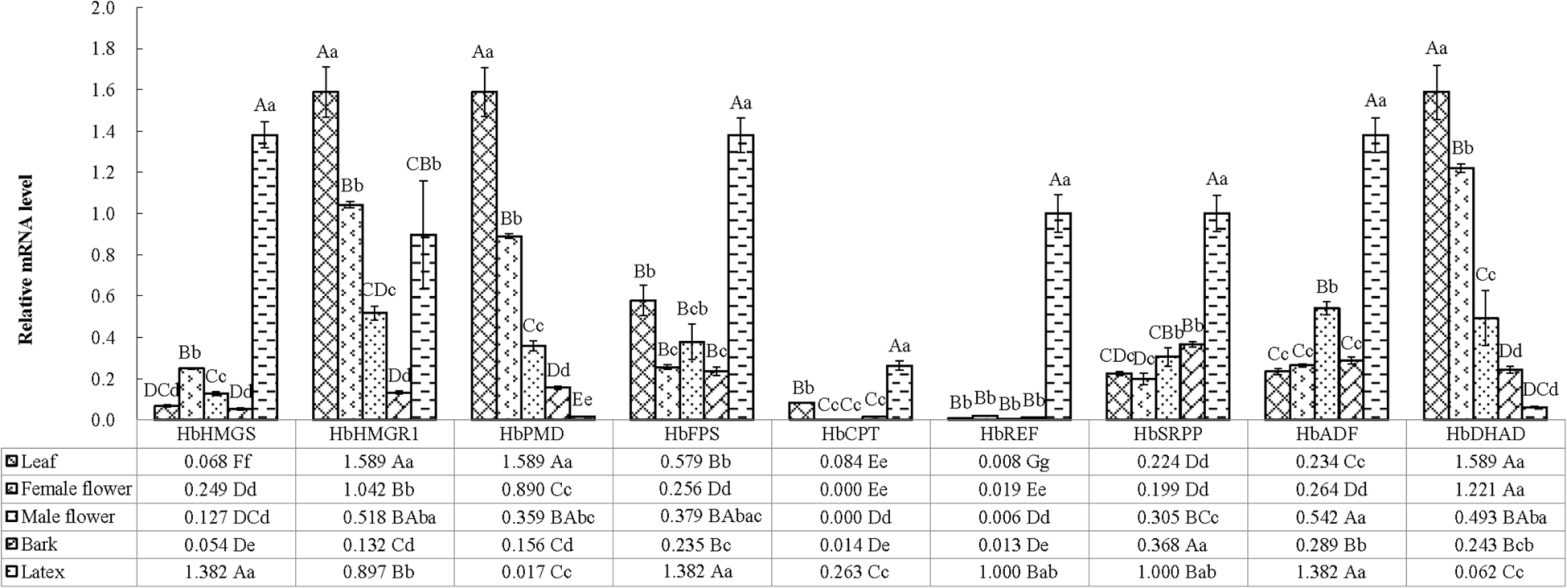
Relative expression profiles of different genes in diverse organs and tissues from *H. brasiliensis* ‘CATAS 73397’ young mature trees. Data are means of three biological replications, and error bars represent standard errors of the means. Shortest significant range (SSR) multiple comparison tests were employed to perform one-way analyses of variance at the 0.01 and 0.05 levels. The different capital and lowercase letters above the bars indicate that the differences in expression values among different tissues for each gene are extremely significant (*P* < 0.01) and significant (*P* < 0.05), respectively. The different capital and lowercase letters in cell grids indicate that the differences in expression values of different genes within the same tissue are extremely significant (*P* < 0.01) and significant (*P* < 0.05), respectively

To explore the expression levels of genes related to latex metabolism in newly tapped young mature rubber trees of the clonal cultivar CATAS 73397, total RNA from leaves, female flowers, male flowers, bark, and latex were analyzed quantitatively by RT-PCR using gene-specific primer pairs. The average expression levels of the nine genes examined in latex tissue were significantly greater than those in male flowers and bark (*P* < 0.05), and the average expression levels of all of the genes in leaves were significant greater than those in bark (*P* < 0.05), but no significant differences were observed among female flowers, male flowers and bark. *HbHMGS*, *HbFPS*, *HbCPT*, *HbREF*, *HbSRPP* and *HbADF* were strongly expressed in latex tissue (Fig. 1), with values of 20.33-, 5.54-, 10.90- and 25.77-fold (*HbHMGS*), 2.39-, 5.39-, 3.64- and 5.89-fold (*HbFPS*), 3.13-, 8685.16-, 17,256.19- and 18.26-fold (*HbCPT*), 125.09-, 52.96-, 158.39- and 79.81-fold (*HbREF*), 4.46-, 5.03-, 3.27- and 2.72-fold (*HbSRPP*) and 5.90-, 5.23-, 2.55- and 4.79-fold (*HbADF*) those in leaves, female flowers, male flowers and bark, respectively (*P* < 0.01). Nevertheless, *HbHMGR1*, *HbPMD* and *HbDHAD* were strongly expressed in leaves, with values of 1.53-, 3.07-, 12.05- and 1.77-fold (*HbHMGR1*), 1.79-, 4.42-, 10.19- and 95.46-fold (*HbPMD*) and 1.30-, 3.22-, 6.53-,and 25.48-fold (*HbDHAD*) those in female flowers, male flowers, bark and latex respectively (*P* < 0.01). Furthermore, the *HbHMGR1*, *HbPMD* and *HbDHAD* mRNA quantities were greatest in the leaf organs (Fig. 1), with quantities that were 23.39-, 2.74-, 18.86-, 198.80-, 7.09- and 6.78-fold (*P* < 0.01) those of *HbHMGS*, *HbFPS*, *HbCPT*, *HbREF*, *HbSRPP* and *HbADF*, respectively. The quantity of *HbDHAD* mRNA was greatest in the female flowers, being 4.90, 1.17, 1.37, 4.76, 40,262.32, 64.66, 6.14 and 4.62 times (*P* < 0.01) those of *HbHMGS*, *HbHMGR1*, *HbPMD*, *HbFPS*, *HbCPT*, *HbREF*, *HbSRPP* and *HbADF*, respectively. The greatest mRNA level in the male flowers was that of *HbADF*, with levels of 4.28, 1.51, 35,534.92, 85.90 and 1.78 times (*P* < 0.05 or *P* < 0.01) those of *HbHMGS*, *HbPMD*, *HbCPT*, *HbREF* and *HbSRPP*, respectively, but the levels were not significantly different between *HbADF* and *HbHMGR1*, *HbFPS* and *HbDHAD* (*P* > 0.05). *HbSRPP* was dominantly expressed in bark, at a level 6.86, 2.79, 2.36, 1.57, 25.50, 29.35, 1.27 and 1.51 times (*P* < 0.01) those of *HbHMGS*, *HbHMGR1*, *HbPMD*, *HbFPS*, *HbCPT*, *HbREF*, *HbADF* and *HbDHAD*, respectively. The *HbHMGS*, *HbFPS* and *HbADF* expression levels were greatest in the latex, with levels 1.54, 83.00, 5.25, 1.38, 1.38 and 22.15 times (*P* < 0.01) those of *HbHMGR1*, *HbPMD*, *HbCPT*, *HbREF*, *HbSRPP*, and *HbDHAD*, respectively. These results imply that the majority of these genes are specifically/preferentially expressed in laticifer cells at the first tapping. As rubber synthetic genes, they are expressed where rubber is synthesized.

### Dynamic expression levels and correlations among genes related to latex metabolism in latex during the incipient periods of tapping

**Fig. 2:**
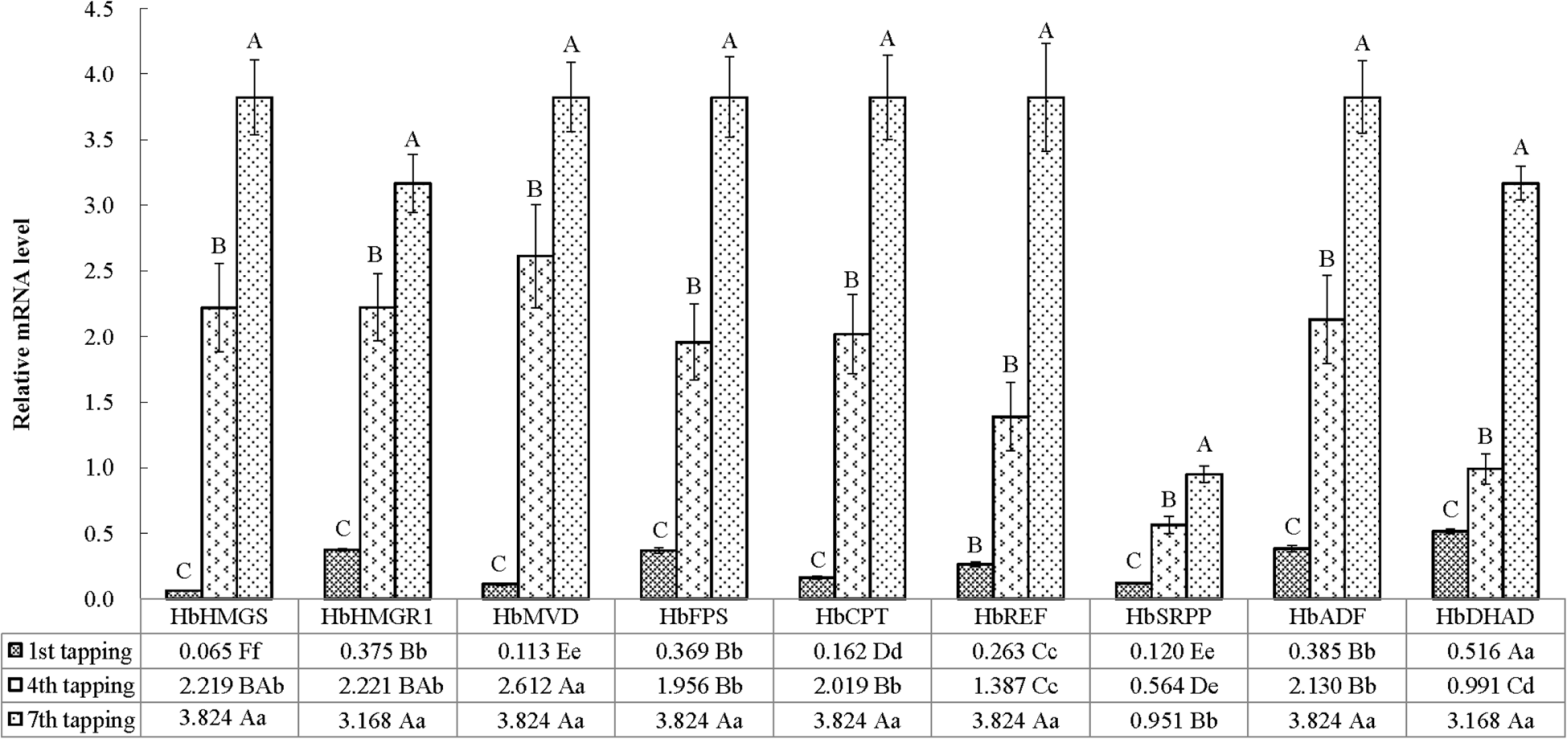
Variations in the mRNA levels of different genes in latex at different tapping times from young newly tapped ‘CATAS 73397’ rubber trees. Different capital superscript letters above the bars denote significant differences among the three tapping times for each gene at the 1% level. Different capital and lowercase letters in cell grids denote significant differences among different genes at the same tapping time at the 1 and 5% levels, respectively

Nine genes implicated in latex metabolism, *HbHMGS*, *HbHMGR1*, *HbPMD*, *HbFPS*, *HbCPT*, *HbREF*, *HbSRPP*, *HbADF* and *HbDHAD*, were all expressed at the 1st, 4th and 7th tappings after opening ‘CATAS 73397’ rubber trees (Fig. 2). The mRNA levels of the nine genes in the 4th and 7th tappings were greater than in the 1st tapping (*P* < 0.01). The expression levels of the nine genes were the greatest at the 7th tapping, with extremely significant values of 12.77 and 1.88 times those at the 1st and 4th tappings, respectively. Thus, we believe that the nine genes were transcribed at low levels in latex before the opening of rubber tree for tapping and a significant increase in their transcript levels was induced after mechanical stimulations. The greatest expression level at the 1st tapping were that of *HbDHAD*, which was 7.96, 4.57, 4.28, 3.18, 1.96, 1.40, 1.38 and 1.34 times those of *HbHMGS*, *HbPMD*, *HbSRPP*, *HbCPT*, *HbREF*, *HbFPS*, *HbHMGR1* and *HbADF*, respectively, which were extremely significant differences (*P* < 0.01). The *HbPMD* expression level was greatest at the 4th tapping, and it was significant or extremely significant at 4.63, 2.64, 1.88, 1.34, 1.29, 1.23, 1.18 and 1.18 times (*P* < 0.05 or *P* < 0.01) higher than those of *HbSRPP*, *HbDHAD*, *HbREF*, *HbFPS*, *HbCPT*, *HbADF*, *HbHMGS* and *HbHMGR1*, respectively. However, at the 7th tapping, differences in mRNA expression levels in the latex were not significant among these genes, after the exclusion of the weakly expressed HbSRPP. Similarly, the latex output per tree per tapping in newly-opened young mature rubber plantings exhibited an increasing tendency as the tapping number increased. Among the three tappings, the greatest latex yield per tree occurred at the 7th tapping, and its 48.33 mL/t was 19.33 and 2.12 times greater than the yields of the 1st and 4th tappings, respectively. A prominent positive correlation occurred between the expression levels of the nine genes in the latex tissues and the tapping time (*R* = 0.893, *P* < 0.01) and the latex yield (*R* = 0.892, *P* < 0.01).

As shown in Table 1, in the 1st, 4th and 7th tappings, an extremely significant correlation was observed between the mRNA expressions of *HbPMD* and *HbHMGR1* in latex tissues, with a correlation coefficient of 1.000 (*P* < 0.01). The dynamic expression of *HbCPT* was significantly positively correlated with in the expression levels of both *HbHMGS* and *HbFPS*, with correlation coefficients of 0.997 and 0.998, respectively (*P* < 0.05). There was an extremely significant positive correlation between the expression level of *HbSRPP* and those of *HbHMGS* and *HbCPT*, with correlation coefficients of 0.999 and 1.000, respectively (*P* < 0.01). The expression changes of *HbADF* mRNA had significantly positive correlations with those of *HbHMGS*, *HbFPS*, *HbCPT* and *HbSRPP*, with correlation coefficients of 0.997, 0.998, 1.000 and 1.000, respectively (*P* < 0.05 or *P* < 0.01).

**Table 1:** Correlation analysis among the expression levels of latex metabolism-related enzyme/protein genes at the initial period of tapping

Genes	*HbHMGS*	*HbHMGR1*	*HbPMD*	*HbFPS*	*HbCPT*	*HbREF*	*HbSRPP*	*HbADF*
*HbHMGR1*	0.995							
*HbMVD*	0.994	1.000**						
*HbFPS*	0.991	0.973	0.97					
*HbCPT*	0.997*	0.985	0.982	0.998*				
*HbREF*	0.957	0.924	0.918	0.987	0.976			
*HbSRPP*	0.999**	0.989	0.987	0.996	1.000**	0.969		
*HbADF*	0.997*	0.985	0.982	0.998*	1.000**	0.976	1.000**	
*HbDHAD*	0.905	0.858	0.851	0.953	0.935	0.989	0.924	0.935

Single (*) and double (**) superscript asterisk marks values of the correlation coefficient significantly different from zero at the 0.05 and 0.01 levels, respectively

### Exogenous phytohormone-induced expression in latex of latex metabolic genes

**Fig. 3:**
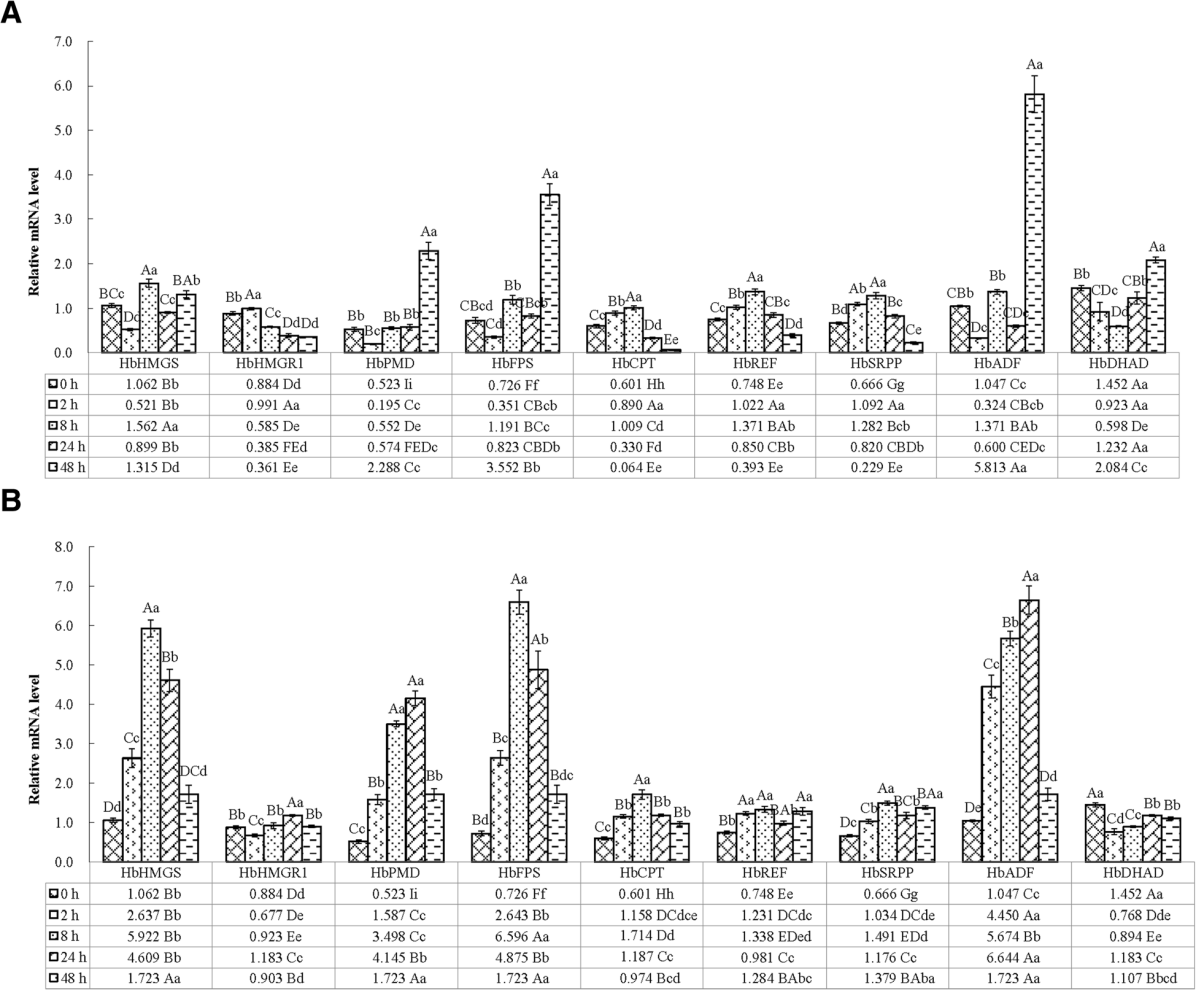
mRNA expression patterns of different genes in latex during the 1st tapping of trees treated with exogenous phytohormones. **a**: Gene expression levels in latex of the trees treated with ethephon; **b**: Gene expression levels in latex of the trees treated with methyl jasmonate. Different lowercase and uppercase letters on top of the bars among different treatment times for each gene indicate significance at the 0.05 and 0.01 levels, respectively. Different capital and lowercase letters in cell grids denote significant differences among different genes within the same treatment time at the 1 and 5% levels, respectively

In comparison to the control 0 h group (tapped 0 h after treatment), the average expression levels of the nine investigated genes in the 50 mg/L ETH-treated plants in the 8- and 48-h groups were high, but the average expression levels in the 2- and 24-h groups were low. Statistically, significant differences were found in the mean expression levels only between the 48-h groups and the control, but not between the 2–24-h groups and the control. In the latex, the average relative expression level of the nine genes in the 48-h group was significant at 2.09, 2.55, and 2.47 times (*P* < 0.05) greater than in the 0-, 2-, and 24-h groups, respectively, but there were no significant differences among the other groups (*P* > 0.05). The expressions levels of *HbPMD*, *HbFPS*, *HbADF* and *HbDHAD* were greatest in the 48-h group (Fig. 3a), with highly significant levels of 4.38, 11.75, 4.14, and 3.99 times (*HbPMD*), 4.89, 10.13, 2.98 and 4.32 times (*HbFPS*), 5.55, 17.92, 4.24 and 9.70 times (*HbADF*) and 1.44, 2.26, 3.49, and 1.69 times (*HbDHAD*) those in the 0-, 2-, 8-, and 24-h groups, respectively (*P* < 0.01). Nevertheless, *HbHMGS*, *HbCPT*, *HbREF* and *HbSRPP* were predominantly expressed in the 8-h group, with values of 1.47, 3.00, 1.74 and 1.19 times (*P* < 0.05 or *P* < 0.01; *HbHMGS*), 1.68, 1.13, 3.06 and 15.75 times (*P* < 0.01; *HbCPT*), 1.83, 1.34, 1.61 and 3.49 times (*P* < 0.01; *HbREF*) and 1.92, 1.17, 1.56 and 5.59 times (*P* < 0.05 or *P* < 0.01; *HbSRPP*) those in the 0-, 2-, 24-, and 48-h groups, respectively. *HbHMGR1* was strongly expressed in the 2-h group, at a value of 1.12, 1.70, 2.57 and 2.75 times (*P* < 0.01) those in the 0-, 8-, 24-, and 48-h groups, respectively. Furthermore, the *HbDHAD* mRNA level was greatest in the control 0-h group (Fig. 3a), with an extremely significant level of 1.37, 1.64, 2.78, 2.00, 2.41, 1.94, 2.18 and 1.39 times (*P* < 0.01) those of *HbHMGS*, *HbHMGR1*, *HbPMD*, *HbFPS*, *HbCPT*, *HbREF*, *HbSRPP* and *HbADF*, respectively. The *HbSRPP* mRNA level was greatest in the 2-h group, with a level that was 2.10, 5.61, 3.12 and 3.37 times (*P* < 0.01) those of *HbHMGS*, *HbPMD*, *HbFPS* and *HbADF*, respectively. There were no remarkable differences between *HbSRPP* and *HbHMGR1*, *HbCPT*, *HbREF* or *HbDHAD*. The greatest mRNA level in the 8-h group was that of *HbHMGS*, being 2.67, 2.83, 1.31, 1.55, 1.14, 1.22, 1.14 and 2.61 times (*P* < 0.05 or *P* < 0.01) those of *HbHMGR1*, *HbPMD*, *HbFPS*, *HbCPT*, *HbREF*, *HbSRPP*, *HbADF* and *HbDHAD*, respectively. The expression level of *HbDHAD* was dominant in the 24-h group, being 1.37, 3.20, 2.15, 1.50, 3.73, 1.45, 1.50 and 2.05 times (*P* < 0.01) those of *HbHMGS*, *HbHMGR1*, *HbPMD*, *HbFPS*, *HbCPT*, *HbREF*, *HbSRPP* and *HbADF*, respectively. The *HbADF* expression level was greatest in the 48-h group, at 4.42, 16.11, 2.54, 1.64, 90.79, 14.77, 25.38 and 2.79 times (*P* < 0.01) those of *HbHMGS*, *HbHMGR1*, *HbPMD*, *HbFPS*, *HbCPT*, *HbREF*, *HbSRPP* and *HbDHAD*, respectively. Pearson’s correlation analysis indicated that there were positive correlations between the expression levels of *HbPMD* and *HbFPS* in latex and the duration of the ETH treatment (*R* = 0.911, *P* < 0.05; 0.894, *P* < 0.05).

The mean expression levels of the nine tested genes in the plants treated with 30 mg/L MeJA for 2–48 h were high in comparison to the control group, while no statistically significant differences were observed in the mean expression levels between the 2- and 48-h groups and the control. In the latex, the mean relative expression level of the nine genes in the 8-h group was a significant 3.64- and 2.24-fold (*P* < 0.05 or *P* < 0.01) greater than the mean levels in the 0- and 48-h groups, respectively, but there were no obvious differences between the 8-h group and the 2- and 24-h groups (*P* > 0.05). The expression levels of *HbHMGS*, *HbFPS*, *HbCPT*, *HbREF* and *HbSRPP* were the greatest in the 8-h group (Fig. 3b), with extremely significant levels of 5.58-, 2.25-, 1.28- and 3.44-fold (*P* < 0.01; *HbHMGS*), 9.08-, 2.50-, 1.35- and 3.83-fold (*P* < 0.05 or *P* < 0.01; *HbFPS*) and 2.85-, 1.48-, 1.44- and 1.76-fold (*P* < 0.01; *HbCPT*) those in the 0-, 2-, 24-, and 48-h groups, respectively. The expressions level of *HbREF* in the 8-h group was a significant 1.79- and 1.36-fold (*P* < 0.05 or *P* < 0.01) greater than its levels in the 0- and 24-h groups, respectively. The expression level of *HbSRPP* in the 8-h group was significantly greater, at 2.24-, 1.44- and 1.27-fold (*P* < 0.05 or *P* < 0.01) than in the 0-, 2-, and 24-h groups. *HbHMGR1*, *HbPMD* and *HbADF* were strongly expressed in the 24-h group, with values 1.34-, 1.75-, 1.28- and 1.31-fold (*P* < 0.01; *HbHMGR1*) and 6.34-, 1.49-, 1.17- and 3.85-fold (*P* < 0.01; *HbADF*) those of the 0-, 2-, 8-, and 48-h groups, respectively. *HbPMD* was expressed strongly in the 24-h group, with a value that was 7.93-, 2.61- and 2.41-fold (*P* < 0.01) those in the 0-, 2-, and 48-h groups, respectively. *HbDHAD* was significantly weakly expressed in the 2–48-h groups, being 1.89-, 1.62-, 1.23- and 1.31-times (*P* < 0.01) lower than in the 0-h group. Additionally, the *HbADF* mRNA level reached a maximum in the 2-h group (Fig. 3b), being extremely significantly greater at 1.69, 6.57, 2.80, 1.68, 3.84, 3.61, 4.31 and 5.80 times (*P* < 0.01) the levels of *HbHMGS*, *HbHMGR1*, *HbPMD*, *HbFPS*, *HbCPT*, *HbREF*, *HbSRPP* and *HbDHAD*, respectively. The *HbFPS* mRNA level was maximal in the 8-h group, with a level that was 1.11, 7.14, 1.89, 3.85, 4.93, 4.42, 1.16 and 7.38 times (*P* < 0.01) those of *HbHMGS*, *HbHMGR1*, *HbPMD*, *HbCPT*, *HbREF*, *HbSRPP*, *HbADF* and *HbDHAD*, respectively. The maximal mRNA level in the 24-h group was that of *HbADF*, at 1.44, 5.61, 1.60, 1.36, 5.60, 6.77, 5.65 and 5.61 times (*P* < 0.01) those of *HbHMGS*, *HbHMGR1*, *HbPMD*, *HbFPS*, *HbCPT*, *HbREF*, *HbSRPP*, *HbADF* and *HbDHAD*, respectively. The maximal expression levels in the 48-h group were those of *HbHMGS*, *HbPMD*, *HbFPS* and *HbADF*, being 1.91, 1.77, 1.34 and 1.56 times (*P* < 0.05 or *P* < 0.01) those of *HbHMGR1*, *HbCPT*, *HbREF* and *HbDHAD*, respectively. However, there were no remarkable differences among the expression levels of *HbHMGS*, *HbPMD*, *HbFPS*, *HbADF* and *HbSRPP*.

### Expression profiles of latex metabolic genes in different clones

To examine the expression patterns of natural latex metabolic enzyme/protein genes, we performed qRT-PCR expression analyses of these genes using high- and medium-yielding cultivated varieties and wild germplasm with low yields. The qRT-PCR analyses of gene expression were carried out in ten varieties and ten wild germplasm for each gene. There were highly significant differences in the expression levels of the genes in latex among the 20 clones (Table 2). The mean expression level analysis in the 20 clones for each gene revealed that *HbSRPP* and *HbADF* had the greatest expression levels, while *HbCPT* and *HbREF* had the lowest expression levels. The expression values of *HbSRPP* and *HbADF* in latex from the 20 clones both were 2.19- and 2.11-fold (*HbSRPP*) and 2.10- and 2.02-fold (*HbADF*; *P* < 0.01) significantly greater than those of *HbCPT* and *HbREF*, respectively. *HbFPS* was expressed at an extremely significant 1.88-fold (*P* < 0.01) greater level than *HbCPT*. *HbFPS* and *HbDHAD* had significant 1.81- and 1.64-fold (*P* < 0.05) greater expression levels than *HbREF*, and *HbHMGR1* was expressed at a significant 1.68-fold (*P* < 0.05) greater level than *HbCPT*. However, there were no obvious differences among the expression levels of the other genes (*P* > 0.05). The expression values of different genes in the same rubber tree clone varied within a specific range, and the change range for the expression values for the nine individual genes in a given clone was 2.48–3353.23 times, with the largest variation in gene expression levels existing in the clone MT/IT/13 29/8. In contrast, expression level fold changes in clone RO/PB/1 2/124 were the smallest. Thus, differential mRNA expressions of genes in high- and medium-yielding varieties and low-yielding wild germplasm varied with the gene characteristics, which caused bigger fluctuations in the average mRNA expression levels in different yielding clones.

**Table 2:** Comparative analysis on expression of different genes in the same clone and of the same gene in different clones

Clones	Gene mRNA levels									
	*HbHMGS*	*HbHMGR1*	*HbPMD*	*HbFPS*	*HbCPT*	*HbREF*	*HbSRPP*	*HbADF*	*HbDHAD*	Average
RRIM 600	0.703 EDde	0.304 HIj	0.328 HGh	0.690 CEDfde	0.390 CFEDgcfde	0.365 DCEed	1.323 Ded	1.313 BECDbcd	0.827 CDcd	0.694 bac
PR 107	0.653 EDFfe	1.186 Bb	0.839 CBDcb	2.144 Aa	0.436 CEDcde	0.880 BAb	0.464 KJkj	1.669 BAba	0.496 KIJHGfhig	0.974 ba
TSF 523	0.989 CBc	0.423 HIij	0.748 CEDcd	0.861 CEBDcde	0.502 CBDcd	1.001 Aa	1.897 Bb	0.944 GFECDHe	0.559 IHGfeg	0.881 bac
TSF 628	0.546 IEHGFfheg	0.475 GHIi	0.633 FEDed	0.402 Ef	0.228 FEghi	0.156 GFHg	2.132 Aa	0.893 GFEIDHef	0.931 CBDcb	0.711 bac
TSF 192	0.411 IHGjhi	0.410 HIij	0.367 HGhg	0.447 EDfe	0.318 FEDghfie	0.913 Aba	0.420 KJkjl	0.924 GFECIDHe	0.847 CDcd	0.562 bac
CATAS 73397	0.540 IEHGFfheg	0.470 GHIi	0.301 Hh	0.749 CEDfde	0.252 FEghfi	0.365 DCEed	1.396 De	0.838 GFEIHefg	0.412 KIJjhi	0.592 bac
CATAS 72059	0.329 Ij	0.286 Ij	0.456 FHGhfg	0.415 EDf	0.155 Fi	0.215 GFEgf	1.295 Dd	0.528 GIHhg	0.609 FHGfe	0.476 c
CATAS 879	0.849 CDdc	0.399 HIij	0.449 FHGhfg	0.974 CBDcd	0.362 CFEDghfde	0.378 DCed	0.380 KJkl	1.792 BAa	1.057 Bb	0.738 bac
CATAS 78426	0.619 EGFfe	0.491 GHih	0.012 Ii	0.844 CEBDcde	0.398 CFEDgcfde	0.303 DFEef	1.612 Cc	1.948 Aa	0.549 IJHGfheg	0.753 bac
CATAS 87662	1.148 Bb	0.819 DEfg	0.934 CBb	1.210 CBcb	0.695 Bb	0.039 Hh	0.827 GHh	1.042 FECDecd	2.378 Aa	1.010 a
RO/PB/1 2/124	0.377 IHjhi	0.837 DEfge	0.554 FEGef	0.869 CEBDcde	0.570 CBcb	0.755 Bc	0.920 GFg	0.428 JIh	0.371 KJji	0.631 bac
MT/IT/13 29/8	0.518 IEHGFfhig	1.086 CBcb	0.640 FEDed	0.357 Ef	0.231 FEghi	0.423 DCd	0.767 IHih	0.000 Ji	0.791 EDd	0.535 bac
MT/C/2 10/49	0.670 EDfe	1.877 Aa	1.543 Aa	0.847 CEBDcde	0.414 CEDcfde	0.878 BAb	0.694 Ii	1.388 BCDbc	0.649 FEGe	0.995 ba
RO/J/6 32/49	0.647 EDFfe	1.000 CDcd	1.000 Bb	0.770 CEDfde	0.504 CBDcd	1.000 Aa	1.083 Ef	0.000 Ji	1.000 CBb	0.778 bac
RO/PB/1 2/78	0.438 IHGFjhig	0.961 CDcde	0.540 FHEGefg	1.373 Bb	1.853 Aa	0.451 DCd	0.452 KJkj	0.563 GFIHhfg	0.988 CBb	0.846 bac
MT/IT/14 30/18	1.371 Aa	0.940 CDEfde	0.023 Ii	1.371 Bb	0.304 FEDghfie	0.159 GFHg	1.154 Ef	1.371 BCDbc	0.777 FEDd	0.830 bac
RO/C/9 23/219	0.358 IHji	0.770 FEg	0.301 Hh	0.605 EDfde	0.159 Fi	0.140 GHgh	0.339 Kl	0.487 IHhg	0.550 IJHGfheg	0.412 c
RO/A/7 25/198	0.840 CDdc	0.618 GFh	0.297 Hh	0.489 EDfe	0.201 FEhi	0.184 GFHg	0.958 Fg	0.001 Ji	0.482 KIJHGfhig	0.452 c
AC/F/7 38/63	0.585 EHGFfeg	0.489 GHih	0.364 HGhg	0.505 EDfe	0.505 CBDcd	0.126 GHgh	0.507 Jj	1.006 GFECDed	0.433 KIJHjhig	0.502 bc
AC/AB/15 54/980	0.539 IEHGFfheg	1.030 CBcd	0.443 FHGhfg	0.713 CEDfde	0.369 CFEDghfde	0.467 Cd	0.750 IHih	1.422 BCb	0.339 Kj	0.675 bac
Mean value	0.656 ± 0.270 YXZwyzx	0.743 ± 0.389 YXZwyvx	0.521 ± 0.370 YZyzx	0.832 ± 0.431 YXwv	0.442 ± 0.361 Zz	0.460 ± 0.324 YZyz	0.968 ± 0.516 Xv	0.928 ± 0.584 Xwv	0.752 ± 0.443 YXZwvx	0.70 ± 0.18
Variation coefficient/%	41.12	52.30	65.62	51.77	81.67	70.36	53.27	62.96	58.85	26.07

Values followed by different uppercase letters ‘ABCDEFGHIJK’ and lowercase letters ‘abcdefghijkl’ within the same column indicate significant difference at 0.01 and 0.05 levels, respectively; values followed by different uppercase letters ‘XYZ’ and lowercase letters ‘vwxyz’ within the same row denote significant difference at 0.01 and 0.05 levels, respectively

Analysis of the average expression levels of multiple genes within the same clone revealed the greatest levels in the clones CATAS 87662, MT/C/2 10/49, and PR 107, and the lowest in the clones RO/C/9 23/219, RO/A/7 25/198 and CATAS 72059. The average expression levels of different genes in clones CATAS 87662, MT/C/2 10/49 and PR 107 were a significant 2.45-, 2.23- and 2.12-fold (CATAS 87662), 2.41, 2.20 and 2.09-fold (MT/C/2 10/49) and 2.36, 2.15 and 2.05-fold (PR 107; *P* < 0.05) greater than those in RO/C/9 23/219, RO/A/7 25/198 and CATAS 72059, respectively (Table 2). In addition, the average expression levels of different genes in clone CATAS 87662 were significantly greater than in clone AC/F/7 38/63 (*P* < 0.05). Nevertheless, no obvious differences were found among the other clones (*P* > 0.05). The expression values of the same gene in different rubber tree clones were different. The change in the amplitude of the expression value of a given gene in the 20 clones ranged from 4.17–6016.20-fold, with the greatest variation in the *HbADF* gene’s expression level, while *HbHMGS* had the least variation. The coefficient of variation for the average expression values of nine genes was 26.07%, which suggests that major differences in the average gene expression levels exists among different clones.

### Comparison of latex yields per tree per tapping from different cultivars in different months

Because the latex yields per tree per tapping from the majority of the wild germplasm were only enough to investigate gene expression, an assessment of their yields was not possible. A monthly dynamic analysis of latex yields per tree per tapping from ten cultivars revealed that the major differences existed among the latex yields of the cultivars in different months (Additional file 2: Table S2). Latex yields per tree per tapping in October and November were significantly greater than those in May, June and July (*P* < 0.05). The latex yield per tree per tapping in September was significantly greater than in July (*P* < 0.05). However, no remarkable differences were observed among the other months (*P* > 0.05). The latex yields per tree per tapping from ten cultivars decreased in the following months: October > November > September > August > June > May > July. Latex yields per tree per tapping from the same cultivar in different months underwent a certain change, the amplitude of which was 1.41–4.98-fold, with the change in ‘CATAS 73397’ being the largest, and the greatest yield being 4.98-fold the lowest value of 21.05 mL latex per tree per tapping. The least amount of change occurred in ‘CATAS 78426’, and the lowest latex yield per tree per tapping decreased to 49.67 mL compared with the greatest yield. Thus, high and moderate harvest-time latex-yielding cultivars are sensitive to the month of harvest. That is, the latex yield per tree per tapping of different yield-level cultivars fluctuated greatly in different months.

There were obvious differences among the latex yields per tree per tapping from different cultivars in the same month (Additional file 2: Table S2). The greatest latex yields per tree per tapping among genotypes in May and June were all ‘TSF 628’, those in August and October came from ‘CATAS 879’, and those in July, September and November came from ‘CATAS 87662’, while all of the lowest latex yields per tree per tapping in May, June, July, August and September came from CATAS 73397, and those in October and November were from ‘PR 107’ and ‘TSF 523’, respectively. There was a significant difference, at the 1% level, between the two cultivars in the same month. The variation coefficients for the monthly mean latex yields per tree per tapping and latex yields per tree per year for the ten rubber tree cultivars were 31.25 and 29.04%, respectively, which demonstrated that great differences existed among the various genotypes.

### Comparison of DRCs in different cultivars and different months

The dynamics of the monthly DRCs of the ten cultivars revealed that there was a greater deviation in DRC among all of the cultivars in different months (Additional file 3: Table S3). The DRC in May was very significantly greater than those in October and November (*P* < 0.01), and significantly greater than that in August (*P* < 0.05). DRCs in June, July and September were all very significantly greater than that in November (*P* < 0.01). The DRC in July was much greater than that in October, which was significant greater than that in November (*P* < 0.05). The DRCs of 10 cultivars decreased gradually in the following order: May > July > September > June > August > October > November. A certain variation appeared among the DRCs of the same cultivars in different months, and the range of variation was 1.15–1.76 times, with the maximum variation occurring in ‘CATAS 72059’, and the greatest content was 1.76 times of the lowest content, at 18.92%. The minimum variation was present in ‘PR 107’, and the lowest content decreased 5.08% compared with the largest value. Thus, the alternate month had a great effect on high, moderate and low DRC-containing cultivars. There was an obvious monthly fluctuation in the DRCs of various cultivars.

DRCs in the same month showed marked differences among different cultivars (Additional file 3: Table S3). The DRCs of ‘CATAS 87662’ in May, June, September and November were the greatest among the different genotypes, while the DRCs of ‘PR 107’ were greatest in July, August and October. The DRCs of ‘TSF 523’ in July, August and October were all the lowest; those of ‘TSF 192’ were the lowest in June and September, and ‘CATAS 879’ and ‘CATAS 72059’ had the lowest DRC values in May and November, respectively. There was a very significant difference between the two cultivars in same month (*P* < 0.01). The variation coefficient for the monthly mean DRC value of the ten rubber tree cultivars was 10.89%, which indicated that the differences in monthly mean DRCs was not negligible among the various genotypes.

### Differential analyses of dry rubber yields per tree per tapping of different cultivars and different months

The monthly changes in the dry rubber yields per tree per tapping of 10 cultivars indicated that their dry rubber yields of all had obvious differences during the different months (Additional file 4: Table S4). The dry rubber yield per tree per tapping in September was extremely significantly greater than the yields in May, June and July (*P* < 0.01), and significantly greater than the yield in August (*P* < 0.05). The dry rubber yield per tree per tapping in October was very significantly greater than the yields in June and July (*P* < 0.01) and significantly greater than the yield in May (*P* < 0.05). The dry rubber yields per tree per tapping in August and November were significantly greater than those in June and July (*P* < 0.05). The dry rubber yields per tree per tapping of ten cultivars decreased as follows: October > November > September > August > May > June > July. Although differences in the monthly tappings’ average dry rubber yield per tree was mainly determined by the latex-producing ability of the *Hevea* trees, this latex-producing ability can be affected by the leaf phenology of the rubber tree. Additionally, daily mean photosynthetically active radiation and total precipitation within a month, as well as temperature and wind levels, during tapping are important factors that influence the monthly tappings’ average dry rubber yield per tree. Dry rubber yields per tree per tapping of the same cultivar showed a certain variance, of 1.33–6.70-fold, among different months, with the greatest variance being that of ‘CATAS 73397′. The greatest dry rubber yield per tree per tapping was 6.70-fold that of the lowest yield, which was 3.89 g. The minimal variance occurred in ‘CATAS 78426′, in which the lowest dry rubber yield per tree per tapping decreased by 11.65 g compared with the greatest value. Thus, high and moderate harvest-time dry rubber yielding cultivars are susceptible to monthly changes. The dry rubber yield per tree per tapping of different yield-level cultivars showed a large fluctuation in various months.

The dry rubber yields per tree per tapping in the same month displayed marked differences among different cultivars (Additional file 4: Table S4). The dry rubber yields per tree per tapping of ‘CATAS 87662’ in May, July, August, September and November were the greatest among the various genotypes, while the greatest yields during June and October were from ‘CATAS 78426’ and ‘CATAS 879’, respectively. The lowest dry rubber yields per tree per tapping in May, June, August and September were from ‘CATAS 73397’, in July from ‘CATAS 72059’, and in October and November from ‘TSF 523’. There were significant differences between cultivars in same month (*P* < 0.01). The variation coefficient of monthly mean dry rubber yields per tree per tapping of the ten rubber tree cultivars was 45.03%, suggesting that the monthly mean dry rubber yields per tree per tapping has a greater variation among different genotypes.

### Differences in dry rubber yields per plant per month from different cultivars

The dynamic change analysis for dry rubber yields per tree per month from 10 cultivars indicated that there were notable differences among different months for all of the cultivars (Additional file 5: Table S5). The dry rubber yield per tree per month in September was extremely significantly greater than the yields in June, July and August (*P* < 0.01) and significantly greater than those in May and November (*P* < 0.05). The dry rubber yield per tree per month in October was very significantly greater than those in June and July (*P* < 0.01) and significantly greater than that in August (*P* < 0.05). The dry rubber yields per tree per month from the 10 cultivars decreased as follows: October > September > November > May > August > July > June. The dry rubber yields per tree per month of the same cultivar in different months had definite variations of 1.33–9.88-fold. The variation in ‘CATAS 73397’ was the greatest, and the greatest yield was 9.88-fold the lowest value at 27.25 g dry rubber per tree per month. The lowest variation occurred in ‘TSF 628’, and the lowest dry rubber yield per tree per month decreased by 108.31 g in comparison with the greatest yield. Thus, high and moderate monthly dry rubber-yielding cultivars are particularly sensitive to the change in month. The dry rubber yields per tree per month of different yield-level cultivars fluctuated with the changing months.

There were apparent differences in monthly dry rubber yields per tree in the same month among different cultivars (Additional file 5: Table S5). ‘CATAS 87662’ had the greatest dry rubber yield per tree per month in May, July, September and November, while ‘CATAS 78426’, ‘TSF 628’ and ‘CATAS 879’ had the greatest yields in June, August and October, respectively. ‘CATAS 73397’ had the lowest dry rubber yield per tree per month for May, June, August and September, while ‘CATAS 72059’, ‘PR 107’ and ‘TSF 523’ had the lowest yields in July, October and November, respectively. There was only a 1% significant difference between cultivars in the same month. The variation coefficients for the monthly average dry rubber yields per tree and the annual dry rubber yields per tree of ten rubber tree cultivars were both 47.12%, which demonstrated that there were conspicuous differences in both yield values among the various genotypes.

### Correlation of latex metabolic gene expression levels in *Hevea brasiliensis* with dry rubber yield and its related traits

Correlation analyses of the relative expression levels of nine genes encoding key regulatory enzymes or proteins in natural latex metabolism in the latex tissues of the mature tapped rubber trees of ten cultivars with the latex yields per tree per tapping, monthly DRC, dry rubber yields per tree per tapping and dry rubber yields per tree per month for the 7 months from May to November 2013, as well as with the annual latex yields per tree and annual dry rubber yields per tree, were performed (Table 3). The correlation analyses indicated that there were significant positive correlations between the *HbHMGS* expression level in latex and latex yields per tree per tapping in August and September, with correlation coefficients of 0.654 and 0.746, respectively (*R*_0.05_ = 0.632; *R*_0.01_ = 0.765). However, a significantly negative correlation existed between the *HbREF* expression level in latex and latex yields per tree per tapping in November, with a correlation coefficient of − 0.682. The expression level of *HbDHAD* in latex was significantly and very significantly positively correlated with latex yields per tree per tapping in August and November, respectively, with correlation coefficients of 0.647 and 0.882, respectively. The expression level of *HbHMGR1* in latex was significantly and very significantly positively correlated with the monthly DRCs from May to November and average monthly DRC, with correlation coefficients of 0.668, 0.646, 0.778, 0.835, 0.707, 0.774, 0.703 and 0.855, respectively. There were significant positive correlations between the *HbFPS* expression level in latex to the monthly DRCs in July and August, as well as the average monthly DRC, with correlation coefficients of 0.634, 0.704 and 0.675, respectively. The *HbCPT* and *HbDHAD* expression levels in latex had significant positive correlations with the monthly DRC in May, with correlation coefficients of 0.664 and 0.764, respectively (*R*_0.05_ = 0.632; *R*_0.01_ = 0.765)*.* The *HbHMGS* expression level in latex showed a significantly positive correlation with dry rubber yields per tree per tapping in September, with a correlation coefficient of 0.648. The *HbCPT* mRNA expression level in latex had a significant positive correlation with dry rubber yields per tree per tapping in September and November, with correlation coefficients of 0.719 and 0.708, respectively (*R*_0.05_ = 0.632; *R*_0.01_ = 0.765). Nevertheless, the *HbREF* expression level in latex demonstrated a significant negative correlation with dry rubber yields per tree per tapping in October, with a correlation coefficient of − 0.668. The *HbDHAD* expression level in latex were significantly or very significantly positively correlated with dry rubber yields per tree per tapping from May to November, except for June, July and October, and the average monthly dry rubber yields per tree per tapping, with correlation coefficients of 0.633, 0.748, 0.770, 0.891 and 0.746, respectively. The *HbCPT* mRNA expression level in latex was positively correlated with the monthly dry rubber yields per tree in September and November, with correlation coefficients of 0.641 and 0.705, respectively (*R*_0.05_ = 0.632; *R*_0.01_ = 0.765). However, the *HbREF* mRNA expression level in latex was negatively correlated with the monthly dry rubber yields per tree in October, with a correlation coefficient of − 0.675. The *HbDHAD* mRNA expression in latex was significantly and highly significantly positively correlated with the monthly dry rubber yields per tree from May to November, except for June, and both the mean monthly and annual dry rubber yields per tree, with correlation coefficients were 0.633, 0.638, 0.667, 0.719, 0.638, 0.875, 0.738 and 0.738, respectively. However, the expression levels of *HbPMD*, *HbSRPP* and *HbADF* in latex were not markedly correlated with dry rubber yield or its related traits, indicating that mevalonate diphosphate decarboxylase and SRPP could not be rate limiting enzymes in *cis*-polyisoprene biosynthesis in rubber tree. Thus, *HbHMGS*, *HbHMGR1*, *HbFPS*, *HbCPT*, *HbREF* and *HbDHAD* may play important roles in the rubber synthesis process. Additionally, the latex and rubber yields are influenced by not only the expression of rubber biosynthesis-related genes in latex but also by the expression of latex drainage-related genes in latex. The *HbREF* mRNA expression level in the high-yield variety CATAS 87662, which has a high DRC, was lower than in variety CATAS 523. This may indicate that the lower expression of *HbREF* in latex is favorable to rubber formation when its DRC is higher, and vice versa, because REF not only plays important roles in rubber biosynthesis but may also be involved in latex coagulation [19, 39].

**Table 3:** Correlation coefficients between the expression of latex metabolism genes in *H. brasiliensis* cultivars with the dry rubber yield and its related traits

Types	Periods	*HbHMGS*	*HbHMGR1*	*HbPMD*	*HbFPS*	*HbCPT*	*HbREF*	*HbSRPP*	*HbADF*	*HbDHAD*
Monthly mean single plant latex yield per tapping and annual latex yield per tree	May	0.365	0.098	0.114	0.031	0.257	−0.399	0.191	0.476	0.413
	June	0.208	−0.051	−0.150	−0.162	0.214	−0.373	0.345	0.42	0.31
	July	0.495	0.136	0.064	0.018	0.482	−0.416	0.288	0.421	0.523
	August	0.654*	−0.044	0.254	−0.019	0.48	−0.354	0.022	0.346	0.647*
	September	0.746*	−0.057	0.417	−0.042	0.606	−0.216	0.264	0.112	0.618
	October	0.174	−0.353	− 0.046	− 0.337	− 0.077	−0.579	− 0.092	0.155	0.472
	November	0.565	0.128	0.254	0.058	0.502	−0.682*	− 0.16	0.244	0.882**
	Monthly mean	0.508	−0.041	0.125	−0.095	0.386	−0.506	0.151	0.358	0.626
	Total annual	0.385	− 0.022	0.110	− 0.161	0.338	−0.601	0.283	0.219	0.629
Dry rubber contents	May	0.403	0.668*	0.546	0.454	0.664*	−0.226	−0.363	− 0.013	0.764*
	June	0.281	0.646*	0.477	0.6	0.378	−0.319	− 0.326	0.081	0.426
	July	0.118	0.778**	0.596	0.634*	0.228	−0.248	−0.232	− 0.024	0.323
	August	0.107	0.835**	0.568	0.704*	0.227	−0.11	−0.351	0.24	0.284
	September	0.446	0.707*	0.410	0.558	0.615	−0.424	−0.107	0.342	0.619
	October	− 0.06	0.774**	0.068	0.6	0.262	0.032	−0.228	0.345	0.047
	November	0.177	0.703*	0.129	0.528	0.519	−0.097	−0.248	0.394	0.367
	Monthly mean	0.266	0.855**	0.453	0.675*	0.521	−0.238	−0.308	0.247	0.502
Dry rubber yields per tree per tapping	May	0.379	0.386	0.258	0.186	0.483	−0.434	0.078	0.342	0.633*
	June	0.241	0.141	−0.061	− 0.022	0.337	− 0.425	0.266	0.406	0.425
	July	0.396	0.324	0.188	0.116	0.484	−0.488	0.199	0.305	0.617
	August	0.502	0.338	0.377	0.186	0.533	−0.438	− 0.03	0.322	0.748*
	September	0.648*	0.336	0.404	0.2	0.719*	−0.371	0.098	0.243	0.770**
	October	0.212	− 0.182	−0.016	− 0.246	0.042	− 0.668*	−0.107	0.216	0.601
	November	0.572	0.446	0.363	0.286	0.708*	−0.518	−0.195	0.184	0.891**
	Monthly mean	0.48	0.301	0.246	0.127	0.548	−0.52	0.053	0.314	0.746*
Monthly and annual dry rubber yields per tree	May	0.379	0.386	0.258	0.186	0.483	−0.434	0.078	0.342	0.633*
	June	0.234	0.16	−0.091	− 0.005	0.355	− 0.432	0.257	0.414	0.422
	July	0.42	0.328	0.171	0.127	0.521	−0.501	0.18	0.313	0.638*
	August	0.401	0.346	0.376	0.174	0.447	−0.376	− 0.014	0.292	0.667*
	September	0.54	0.357	0.383	0.191	0.641*	−0.355	0.094	0.224	0.719*
	October	0.284	−0.187	0.038	−0.218	0.08	−0.675*	− 0.156	0.174	0.638*
	November	0.549	0.458	0.359	0.286	0.705*	−0.495	−0.182	0.184	0.875**
	Monthly mean	0.458	0.312	0.238	0.131	0.542	−0.517	0.05	0.31	0.738*
	Total annual	0.458	0.312	0.238	0.131	0.542	−0.517	0.05	0.31	0.738*

Single (*) and double (**) superscript asterisk marks values of the correlation coefficient significantly different from zero at the 5 and 1% levels, respectively. The same below

### Mutual correlations among latex metabolic gene expression levels in *H. brasiliensis*

To determine whether there is a synergic interaction among the different enzyme/protein genes related to the synthesis and metabolism of rubber latex that aids in the co-regulation of rubber synthesis and accumulation, the correlations among their expression levels were analyzed. As shown in Table 4, the dynamic expression of *HbPMD* had significant or very significant positive correlations with the expression levels of *HbHMGR1* and *HbREF*, with correlation coefficients of 0.659 and 0.527, respectively (*R*_0.05_ = 0.444; *R*_0.01_ = 0.562). A significant positive correlation in expression levels was observed between *HbFPS* and *HbADF*, with a correlation coefficient of 0.450. These results suggest the cooperative regulation of every gene involved in rubber accumulation during the latex metabolic process.

**Table 4:** Correlation coefficients among the expression levels of latex metabolism-related enzyme/protein genes in rubber tree clones

Types	Genes	*HbHMGS*	*HbHMGR1*	*HbPMD*	*HbFPS*	*HbCPT*	*HbREF*	*HbSRPP*	*HbADF*
Clones	*HbHMGR1*	0.066							
	*HbPMD*	0.008	0.659**						
	*HbFPS*	0.391	0.383	0.176					
	*HbCPT*	−0.039	0.199	0.164	0.435				
	*HbREF*	− 0.152	0.35	0.527*	0.221	0.122			
	*HbSRPP*	0.187	−0.376	−0.112	−0.249	− 0.245	− 0.067		
	*HbADF*	0.31	−0.005	−0.102	0.450*	−0.042	− 0.016	0.017	
	*HbDHAD*	0.438	0.011	0.298	0.183	0.283	−0.232	−0.065	0.015
Cultivated variety	*HbHMGR1*	0.308							
	*HbPMD*	0.543	0.603						
	*HbFPS*	0.415	0.909**	0.5					
	*HbCPT*	0.894**	0.509	0.516	0.546				
	*HbREF*	−0.038	0.192	0.138	0.297	0.085			
	*HbSRPP*	−0.053	−0.384	− 0.147	−0.467	− 0.222	−0.239		
	*HbADF*	0.272	0.338	−0.243	0.54	0.324	0.08	−0.315	
	*HbDHAD*	0.631	0.222	0.498	0.087	0.655*	−0.48	−0.259	− 0.058
Wild germplasm	*HbHMGR1*	0.034							
	*HbPMD*	−0.281	0.811**						
	*HbFPS*	0.383	0.208	−0.089					
	*HbCPT*	−0.271	0.024	0.081	0.62				
	*HbREF*	−0.259	0.63	0.827**	0.109	0.163			
	*HbSRPP*	0.662*	0.061	−0.02	0.164	− 0.328	0.307		
	*HbADF*	0.33	0.364	0.024	0.407	−0.007	−0.106	−0.101	
	*HbDHAD*	0.185	0.269	0.258	0.442	0.467	0.295	0.138	−0.299

In high- and medium-yielding varieties, the changes in *HbCPT* mRNA expression levels were significantly or extremely significantly positively correlated with the expression levels of *HbHMGS* and *HbDHAD*, with correlation coefficients of 0.894 and 0.655, respectively (*R*_0.05_ = 0.632; *R*_0.01_ = 0.765). The dynamic expression of *HbFPS* had an extremely significant positive correlation with in the expression of *HbHMGR1*, with a correlation coefficient of 0.909. In low-yielding wild germplasm, there was an extremely positive correlation between the expression level of *HbPMD* and the levels of *HbHMGR1* and *HbREF*, with correlation coefficients of 0.811 and 0.827, respectively (*R*_0.05_ = 0.632; *R*_0.01_ = 0.765). A marked correlation was observed between the mRNA expression levels of *HbHMGS* and *HbSRPP* in latex tissue, having a correlation coefficient of 0.662. Thus, the synergistic effects among different genes in diverse yield-level cultivated cultivars and wild germplasm may cause differences in the rubber yield and its relative traits.

## Discussion

### Variation in the expression of latex metabolic genes in the CATAS 73397 clone under typical stimulation conditions

Rubber latex of *H. brasiliensis* is essentially the cytoplasm of laticifers (Chow et al., 2011); therefore, latex metabolism and its regenerative capacity are important factors determining rubber yield. In rubber production, the application of an appropriate level of ETH can greatly improve the latex yield of rubber trees. Bark tapping-related damage and exogenous ETH and MeJA stimulation result in the expressional changes of many genes in the laticiferous cells of rubber trees, and they especially induce the up-regulated expression of latex metabolism-related genes that are involved in latex production and drainage. To increase the overall understanding of the functions of latex production- and drainage-related genes, the transcriptional levels of *HbHMGS*, *HbHMGR1*, *HbPMD*, *HbFPS*, *HbCPT*, *HbREF*, *HbSRPP*, *HbADF* and *HbDHAD* in the leaves, male and female flowers, trunk bark and latex from the mature virgin rubber clonal trees of CATAS 73397 were determined by qRT-PCR. The expression levels of latex metabolic genes fluctuated considerably among different tissues from newly tapped young clonal trees of CATAS 73397. Among these, *HbHMGS*, *HbFPS*, *HbCPT*, *HbREF*, *HbSRPP* and *HbADF* were maximally expressed in latex, while *HbPMD* and *HbDHAD* were minimally expressed in latex and *HbHMGR1* was moderately expressed in latex. Furthermore, SRPP was expressed in all tissues rather than being restricted to laticifers in a manner similar to that of dandelion SRPP [40] but different from that of *HbREF*.

Using qRT-PCR, we also determined the transcript levels of nine genes in latex from newly tapped plants under regular bark tappings and plants treated with exogenous ETH and MeJA. The expression levels of the nine genes (encoding critical enzymes or proteins associated with natural latex metabolism) in latex from young newly tapped trees were all inducible at least at one time point, with the exception of *HbDHAD* expression in plants treated with exogenous MeJA, and the expression levels varied based on the stimuli, the induction duration and the gene type, revealing that the up-regulation of the nine genes might be related to the latex production and drainage of rubber trees and beneficial to the systematic defense in laticifers against plant injury.

The expression levels of the nine genes were distinctly elevated in latex tissues in the trunks of the clonal rubber trees of ‘CATAS 73397’ without ETH treatment after the occurrence of mechanical wounding (excluding the first tapping), which suggests that the induced expression of these genes may be a form of self-protection in the tree body. The expression levels of the nine genes had positive correlations with the number of tappings (*P* < 0.01) and latex yield (*P* < 0.01), which was in agreement with the observation that, to some extent, the strength of the response to mechanical damage was positively correlated with the degree of damage. The expression levels of these genes were not only up-regulated after the 7th tapping, but also their expression values, with the exception of HbSRPP, were similar.

The expression levels of *HbPMD, HbFPS, HbADF* and *HbDHAD* were strongly induced after a treatment with 50-mg/L ETH for 48 h, which is the later stage of the treatment. A 50-mg/L ETH treatment induced the maximal expression level of *HbHMGR1*, as well as *HbHMGS, HbCPT, HbREF* and *HbSRPP*, at 2 and 8 h, respectively, which represented the early-middle stages of treatment. However, the strong induction of *HbHMGS, HbFPS, HbCPT, HbREF* and *HbSRPP*, as well as *HbHMGR1, HbPMD* and *HbADF*, after treatment with 30-mg/L MeJA appeared at 8 and 24 h, respectively, which represented the middle-later stage of the treatment. Although the high expression levels of *HbHMGS, HbCPT, HbREF* and *HbSRPP* induced by ETH and MeJA both occurred at 8 h after treatment, the sensitivity and speed of the defense responses of these genes to exogenous MeJA are closer to each other than to those of externally applied ETH. Moreover, there were significant positive correlations between the expression levels of both *HbPMD* and *HbFPS* with the duration of the ETH treatment (*P* < 0.05). Our results support the natural rubber production standard that regular bark tapping should not be allowed to begin before ETH applications to the tapping panel have been sustained for at least 24 h.

Together with tissue-specific expression levels, these results suggest that, except HbDHAD, which was absolutely unresponsive to externally applied MeJA stimuli, the expression levels of the examined genes were all increased after regular bark tapping or exogenous ETH and MeJA treatments, regardless of whether their expression levels in the latex of the mature virgin rubber trees were higher, lower or moderate compared with other tissues that were analyzed. Our data corroborate that the mRNA expression levels of *HbREF* and *HbSRPP* are highly expressed in the laticifer latex in *H. brasiliensis* and can be stimulated by mechanical wounding (tapping) and ETH treatments (Berthelot et al., 2014a).

### Dynamic changes in the rubber yield and yield-related traits during different tapping months

Dynamic trends of yield per tree and the relative characteristics of rubber from May to November after tapping were determined for 10 rubber tree cultivars that showed extremely significant differences in dry rubber yields and the yield-related characteristics. There were significant monthly changes in the rubber yield and its related characteristics. Furthermore, the correlation analysis revealed that the number of tapping months was highly positively correlated with the latex yield and dry rubber yield of individual plants per tapping, with correlation coefficients of 0.929 and 0.889 (*R*_0.05_ = 0.755; *R*_0.01_ = 0.875), respectively, and extremely negatively correlated with the monthly dry rubber content, having a correlation coefficient of − 0.886, but not with the monthly dry rubber yield of single plant, which had a correlation coefficient of 0.732. The reason for the lack of correlation may be that the tapping times of some cultivars in different months varied from those of other cultivars. Therefore, on the whole, the rubber accumulated gradually with an increase in tapping months, which was consistent with the local phenomenon of rush-harvesting rubber for 3 months, from approximately September to November in Hainan.

### Monthly changes in rubber yield and its related traits among genotypes of *H. brasiliensis*

The monthly mean latex yields per tree per tapping of the 10 varieties ranged from 61.74 to 162.80 mL, with a variation coefficient of 12.46–52.59%. The monthly mean dry rubber contents of the ten varieties fluctuated from 26.61 to 36.87%, with a variation coefficient of 5.95–18.81%. The monthly mean dry rubber yields per tree per tapping of the ten varieties varied from 14.27 to 60.81 g, with a variation coefficient of 10.28–68.72%. The monthly average dry rubber yields per tree of the ten varieties ranged from 112.02 to 507.75 g, with a variation coefficient of 12.88–74.87%. Thus, there were evident differences in the monthly changes in the rubber yield and its related traits during various harvest periods among the ten different rubber tree varieties.

### Differential gene expression profiles in the latex metabolic pathway and their relevance to dry rubber yield and yield-related traits

The expression levels of most genes involved in the latex metabolic pathway varied greatly in the 20 clones, and *HbCPT*, which had a variation coefficient of 81.67%, exhibited the greatest degree of variation. Predominant expression levels of *HbHMGS*, *HbPMD*, *HbCPT* and *HbHMGR1* were observed in wild germplasm irrespective of the dry rubber yield of the clone, which could indicate that there are a number of key genes and interacting networks of small-effect genes that affect the very complicated quantitative trait of rubber yield.

The activity levels of HMGS and HMGR from rubber tree present in latex are positively correlated with the DRC of latex [41, 42]. Additionally, *HbHMGS1* and *HbHMGR1* seem to be cooperatively modulated during rubber synthesis [43, 44]. Here, the level of *HbHMGR1* expression in latex had a marked positive correlation with the monthly DRC, with the exception of October, and the average monthly DRC, which was not the case for *HbHMGS*. However, the expression level of *HbHMGS* in latex showed significant positive correlations with the latex yields per tree per tapping in August and September, and dry rubber yields per tree per tapping in September. Moreover, the cooperative expression of *HbHMGS* and *HbHMGR1* was not conspicuous in the latex of newly tapped young *H. brasiliensis* clone CATAS 73397 in the 1st, 4th and 7th tappings (Table 1), but the expression level of *HbHMGS* in latex showed significant positive correlations with those of *HbCPT*, *HbSRPP* and *HbADF* (*P* < 0.05 or *P* < 0.01), and the expression level of *HbHMGR1* in latex showed a significant positive correlation with that of *HbPMD* (*P* < 0.01). Thus, we speculated that the regulatory mechanisms of *HbHMGS* and *HbHMGR1* in rubber synthesis may involve increasing the latex yield and DRC, respectively, so as to increase the dry rubber yield.

*HbPMD* expression in the latex of newly tapped young *H. brasiliensis* trees from clone PR 107 at the 1st and 7th tappings after tree opening for tapping [25] and its expression in latex from this clone when it reached the second tapping year were all greater than in ‘RRIM 600’, ‘CATAS 73397’, ‘CATAS 72059’ or ‘CATAS 879, but there was no close correlation among the *HbPMD* gene expression levels. *HbFPS1*’s expression level in latex of the initially tapped young *H. brasiliensis* trees of ‘PR 107’ at the first tapping after opening of trees and *HbFPS*’s expression in the latex of tapped *Hevea* trees from the same clone during its second tapping year were all greater compared with in ‘RRIM 600’, ‘CATAS 73397′, ‘CATAS 72059′ or ‘CATAS 879′, and there was a correlation among *HbFPS* expression levels (*R* = 0.898, *P* < 0.05), which was not the case for *HbFPS1*’s expression at the 7th tapping or *HbFPS2*’s expression at the 1st and 7th tappings [34]. Thus, the dynamic changes of rubber synthesis-related gene mRNA expression levels varied with the clonal varieties, as well as the tapping times and genes.

CPT and REF on the rubber particle’s surface are both essential enzymes for the synthesis of rubber latex [7, 45–47]. In addition, CPTs are also expressed predominantly in laticifers. Aoki et al. described that the transcriptional level of *HbCPT* in latex was 80-fold lower than that of *HbREF* [15]. Nevertheless, the levels of *HbREF* expression in the latex of ‘PR107’, ‘TSF 523’, ‘TSF 192’, ‘CATAS 73397’, ‘CATAS 72059’, ‘RO/PB/1 2/124’, ‘MT/IT/13 29/8’, ‘MT/C/2 10/49’ and ‘RO/J/6 32/49’ clones were only 2.02-, 1.99-, 2.87-, 1.45-, 1.39-, 1.04-, 1.32-, 1.83-, 2.12- and 1.98-fold (*P* < 0.05 or *P* < 0.01), respectively, greater than *HbCPT*. Furthermore, there was no evident difference between the expression levels of *HbCPT* and *HbREF* in the latex of ‘RRIM 600’, ‘TSF 628’, ‘CATAS 879’, ‘CATAS 78426’, ‘RO/C/9 23/219’, ‘RO/A/7 25/198’ and ‘AC/AB/15 54/980’, and the expression levels of *HbREF* were a significant 17.88-, 4.11-, 1.91- and 4.01-fold (*P* < 0.01) lower than those of *HbCPT* in the ‘6–62’, ‘RO/PB/1 2/78’, ‘MT/IT/14 30/18’ and ‘AC/F/7 38/63’ clones, respectively. The different results of the two studies are presumably due to the testing of different *HbREF* family members and different experimental techniques for detecting *HbCPT* and *HbREF* expression levels. Aoki et al. [15] used the rubber elongation factor (GenBank accession number X56535) and cDNA library sequencing, whereas a new rubber elongation factor gene *HbREF* (GenBank accession number MF361124) cloned in our laboratory and real-time qRT-PCR were used in the present study. Previously, *REF* mRNA transcript levels in high-yielding clones were a significant three- to four-fold higher than in low-yield clones, and the *REF* gene expression pattern had a positive correlation with latex (rubber) yield [48]. However, in this study, *REF* mRNA transcripts were not relatively more abundant in high-yielding clones than in low-yield clones, and the *HbREF* expression levels in 20 clones with contrasting yields showed no correlations with latex yield, except for latex yields per tree per tapping in November. The different results of the two studies may be due to the selection of different experimental materials with different growth years and different tapping frequencies for assessing *HbREF* expression levels of different *HbREF* family members. Priya et al. [48] used 18-year-old regularly tapped trees that were subjected to tapping every 2 d with a GenBank KX179469 sequence, while young tapped trees were subjected to tapping every 3 d with a GenBank MF361124 sequence in the present study. Similarly, no correlation was found between the *SRPP* gene expression pattern and latex (rubber) yield. *HbREF* and *HbSRPP* expression levels in the tested clones with contrasting yields may not have correlate with rubber accumulation of rubber because these families have many members that are involved in rubber synthesis and linked on rubber particles [19]. Nonetheless, the induction of both *REF* and *SRPP* by regular tapping after the opening of young mature rubber trees of clone CATAS 73397 correlated with increased natural rubber synthesis, and *REF* and *SRPP* gene expression levels were also induced by both ETH and MeJA treatments.

During the latex flow of mature rubber trees, actin was intercepted at the ends of the cut laticifers and participated in forming proteinaceous networks having microfilament skeletons. The laticifer blocking and wound healing of tapped *H. brasiliensis* can be realized by the proteinaceous network. Finally, they cease the latex flow of rubber trees [49, 50]. Actin depolymerizing factor (ADF) exists widely in eukaryotes and is an important actin-binding protein. Because the equilibrium processes of the actin cytoskeleton are critical for its function, ADF may play a role in the plugging process of the laticifer by modulating actin filament polymerization and depolymerization to regulate the dynamic assembly of the actin cytoskeleton [28]. qRT-PCR analysis in this study showed that not only is *HbADF* predominantly expressed in latex compared with the other eight genes, but also the expression level of this gene varied considerably in the different clones, suggesting that it is involved in modulating the latex drainage of rubber trees.

Isoprenoids are formed by the polymerization of so-called “isoprene activating molecule” IPP. IPP is synthesized from acetyl CoA through acetoacetyl-CoA and mevalonate [25, 51] and is also produced by leucine deamination and transformation to form 3-hydroxy-3-methylglutaryl coenzyme A [21]. In plant cells, pyruvate is not only the principal precursor of alanine, valine and leucine, but it is also the intermediate molecular of IPP and polyisoprene synthesis. Furthermore, dihydroxy-acid dehydratase is the third most common enzyme of the biosynthetic pathway forming branched-chain amino acids, including isoleucine, valine and leucine. In this research, the expression level of *HbDHAD* in latex was significantly positively correlated to different degrees and extents with the latex yield per tree per tapping, average monthly latex yield per tree per tapping, monthly DRC, monthly dry rubber yield per tree and mean monthly and annual dry rubber yields per tree. Thus, the gene might be essential for rubber biosynthesis in *H. brasiliensis*.

## Conclusions

Several clones were used to evaluate the production potential of rubber trees with different origins and genetic backgrounds. We selected the mature virgin rubber clonal trees of CATAS 73397 treated with regular bark tapping, exogenous ETH and MeJA, as well as untreated trees, and investigated the expression levels of nine genes involved in latex metabolism in these clones. Several laticifer-specific genes were involved in the MVA pathway of rubber synthesis or latex metabolism, such as IPP synthesis. In this study, we demonstrated that *HbHMGS*, *HbFPS*, *HbCPT*, *HbREF*, *HbSRPP* and *HbADF* were strongly expressed in latex, while *HbHMGR1*, *HbPMD* and *HbDHAD* were strongly expressed in leaves. Moreover, the nine genes’ expression levels in latex during the 1st, 4th and 7th tappings were positively correlated with the times of the tappings and the latex yield in ‘CATAS 73397′. Both bark tapping and ETH and MeJA stimulation could significantly increase the transcript levels of *HMGS*, *HMGR*, *PMD*, *FPS*, *CPT*, *REF*, *SRPP*, *DHAD* and *ADF*, which showed their greatest expression levels during the last stage of incipient tapping after tree opening. Under ETH and MeJA treatments, the greatest gene expression levels occurred in the later and early-middle stages of treatment and in the early-middle stages of treatment, respectively. A few significant variations in gene expression existed among different genotypes of *H. brasiliensis*. Although these are initial studies on the correlations of latex metabolism-related gene expression levels with rubber yield and yield-related traits, the study advances our knowledge of these genes and how they are expressed in both high-and medium-yield rubber tree varieties and low-yield wild rubber tree germplasm.

## Additional files

Additional file 1: **Table S1.** Primers designed from EST sequences for RT-qPCR analysis of rubber synthetic and latex metabolic genes and their reference gene. (DOC 33 kb)

Additional file 2: **Table S2.** Change analysis of monthly mean latex yield per tapping and annual latex yield from single plant of different cultivars. (DOC 34 kb)

Additional file 3: **Table S3.** Variations in dry rubber contents of different cultivars in the same month and of the same cultivar in different months. (DOC 33 kb)

Additional file 4: **Table S4.** Variability in dry rubber yield per tree per tapping from different cultivars and different harvest months. (DOC 33 kb)

Additional file 5: **Table S5.** Diversity in monthly and annual dry rubber yields of individual plant of different cultivars. (DOC 35 kb)

## Abbreviations

ADF: Actin depolymerizing factor
CPT: *Cis*-prenyltransferase
DHAD: Dihydroxyacid dehydratase;
DRC: Dry rubber content
ETH: Ethephon
FPP: Farnesyl diphosphate
FPS: Farnesyl diphosphate synthase
GPP: Geranyl diphosphate
HMG-CoA: 3-hydroxy-3-methylglutaryl-CoA
HMGR: 3-hydroxy-3-methylglutaryl (HMG)-CoA reductase
HMGR: HMG-CoA reductase
HMGS: 3-hydroxy-3-methylglutaryl (HMG)-CoA synthase
HMGS: HMG-CoA synthase
IPP: Isopentenyl diphosphate
MeJA: Methyl jasmonate
MEP: Methyl-erythritol 4-phosphate
MVA: Mevalonate
PMD: Diphosphomevalonate decarboxylase
REF: Rubber elongation factor
RT-qPCR: Real-time quantitative PCR
SRPP: Small rubber particle protein
