# Supplementary material for: The relationship between latex metabolism gene expression with rubber yield and related traits in Hevea brasiliensis
Source: BMC Genomics. 2018 Dec 10;19:897. doi: 10.1186/s12864-018-5242-4 (PMC6288877; doi:10.1186/s12864-018-5242-4)
Supplement: Supplementary file 1 — Table S1. Primers designed from EST sequences for RT-qPCR analysis of rubber synthetic and latex metabolic genes and their reference gene. (DOC 33 kb) [file 12864_2018_5242_MOESM1_ESM.doc]

**Supporting Information**

**Table S1:** Primers designed from EST sequences for RT-qPCR analysis of rubber synthetic

and latex metabolic genes and their reference genes.

| Primer | GenBank accession No. | Primer sequence (5ʹ→3ʹ) |
| --- | --- | --- |
| HbHMGS-F  HbHMGS-R  HbHMGR1-F  HbHMGR1-R  HbPMD-F  HbPMD-R  HbFPS-F  HbFPS-R  HbCPT-F  HbCPT-R  HbREF-F  HbREF-R  HbSRPP-F  HbSRPP-R  HbADF-F  HbADF-R  HbDHAD-F  HbDHAD-R  18S-F  18S-R | AF429389  AF429388  MF361123  KP677545  AB294716  MF361124  HQ640231  HQ268020  MF361125  AY496880 | 5ʹ-AGAGGCGTAGAGAAATGGC-3ʹ  5ʹ-AATCCTGTCCAAGTCCAATG-3ʹ  5ʹ-TACCATTATTGCCGCACC-3ʹ  5ʹ-GACCTCCTTGTCATCCTCTG-3ʹ  5ʹ-TTCAACCGCATCATCACC-3ʹ  5ʹ-GCCATTACAGCAAGCAACC-3ʹ  5ʹ-GGTTGGTGTATTGAATGGC-3ʹ  5ʹ-ATAGTATGCCTTCCCTCGG-3ʹ  5ʹ-TATGGCATCCTAACCCAGG-3ʹ  5ʹ-TCCAGCCTTATGACCACCT-3ʹ  5ʹ-CAAACTTCAACGACTGCG-3ʹ  5ʹ-GGGTATCTTCCTCTTCATCATC-3ʹ  5ʹ-CGATACTATTGAGAATGTGGTGAAG -3ʹ  5ʹ-CATCTAGGCTAGTGACCGATACATC -3ʹ  5ʹ-TTCATCATCACCACCACTTC-3ʹ  5ʹ-AAGCGATAGGTCCTCTTCG-3ʹ  5ʹ-TGCCAACCCAACCAAAGTC-3ʹ  5ʹ-ACAGGCTGCGATTGTGCTAC-3ʹ  5ʹ-GCTCGAAGACGATCAGATACC-3ʹ  5ʹ-TTCAGCCTTGCGACCATAC-3ʹ |
