# Supplementary material for: The relationship between latex metabolism gene expression with rubber yield and related traits in Hevea brasiliensis
Source: BMC Genomics. 2018 Dec 10;19:897. doi: 10.1186/s12864-018-5242-4 (PMC6288877; doi:10.1186/s12864-018-5242-4)
Supplement: Supplementary file 2 — Table S2. Change analysis of monthly mean latex yield per tapping and annual latex yield from single plant of different cultivars. (DOC 34 kb) [file 12864_2018_5242_MOESM2_ESM.doc]

**Supporting Information**

**Table S2:** Change analysis of monthly mean latex yield per tapping and annual latex yield from single plant of different cultivars.

| Cultivars | Monthly mean latex yield per tree per tapping (ml) | | | | | | | | ml·t^-1^·a^-1^ |
| --- | --- | --- | --- | --- | --- | --- | --- | --- | --- |
|  | May | June | July | August | September | October | November | Average |  |
| RRIM 600  PR 107  TSF 523  TSF 628  TSF 192  CATAS 73397  CATAS 72059  CATAS 879  CATAS 78426  CATAS 87662  Mean value  Variation coefficient/% | 91.72 BCbcd  84.18 BCDecd  80.59 BCDed  148.18 Aa  68.11 CDefd  42.11 Df  58.57 CDef  122.60 BAba  121.91 BAba  113.02 BAbc  93.10 + 32.97 yz  35.42 | 117.43 BAb  58.83 BCDced  71.91 BCDcbd  175.45 Aa  80.85 BCDcbd  21.05 De  33.97 CDed  95.75 BCcb  171.58 Aa  116.76 BAb  94.36 + 52.22 yz  55.34 | 81.77 BCcb  62.30 BCcbd  86.11 BCb  147.93 Aa  62.56 BCcbd  38.99 BCcd  28.90 Cd  98.42 BAb  157.43 Aa  149.24 Aa  91.37 + 46.43 z  50.82 | 109.82 BACbc  84.39 BCdc  125.31 BAbac  142.14 BAba  93.46 BCdc  60.05 Cd  88.44 BCdc  166.64 Aa  126.94 BAbac  158.85 Aa  115.60 + 34.52 yxz  29.86 | 154.02 BAbac  98.16 BCed  179.56 Aba  164.26 BAba  102.81 BCedc  64.40 Ce  105.08 BCedc  152.39 BAbac  133.21 BACbdc  190.24 Aa  134.42 + 40.59 yx  30.20 | 150.65 BCDbcd  76.84 De  96.38 CDed  200.79 BAba  128.35 BCDecd  100.74 CDed  143.22 BCDbcd  249.11 Aa  131.99 BCDecd  169.36 BCbc  144.74 + 51.64 x  35.67 | 147.74 BCcbd  112.34 Cd  100.42 Cd  160.86 BACcb  114.84 BCcd  104.86 Cd  116.44 BCcd  176.44 BAb  143.77 BCcbd  218.99 Aa  139.67 + 37.76 x  27.03 | 121.88 BACbdac  82.44 BCde  105.75 BACbdec  162.80 Aa  93.00 BCdec  61.74 Ce  82.09 BCbdec  151.62 BAbac  140.98 BAba  159.50 Aa  116.18 + 36.31  31.25 | 7070.94 BACdc  4798.77 DCef  5621.97 BDCed  9429.42 Aa  5378.82 BDCed  3732.38 Df  5407.87 Def  6935.97 BAbc  8218.61 Abac  9152.00 Aba  6574.68 + 1909.14  29.04 |

ml·t^-1^·a^-1^: latex yield at milliliter per tree per year.

Values followed by different uppercase letters ‘ABCD’ and lowercase letters ‘abcdef’ within the same column indicate significant difference at 0.01

and 0.05 levels, respectively; values followed by different lowercase letters ‘xyz’ within the same row denote significant difference at 0.05 level.
