# Supplementary material for: The relationship between latex metabolism gene expression with rubber yield and related traits in Hevea brasiliensis
Source: BMC Genomics. 2018 Dec 10;19:897. doi: 10.1186/s12864-018-5242-4 (PMC6288877; doi:10.1186/s12864-018-5242-4)
Supplement: Supplementary file 3 — Table S3. Variations in dry rubber contents of different cultivars in the same month and of the same cultivar in different months. (DOC 33 kb) [file 12864_2018_5242_MOESM3_ESM.doc]

**Supporting Information**

**Table S3:** Variations in dry rubber contents of different cultivars in the same month and of the same cultivar in different months.

Values followed by different uppercase letters ‘ABCDE’ and lowercase letters ‘abcd’ within the same column indicate significant

| Cultivars | Monthly mean dry rubber content (%) | | | | | | | |
| --- | --- | --- | --- | --- | --- | --- | --- | --- |
|  | May | June | July | August | September | October | November | Average |
| RRIM 600  PR 107  TSF 523  TSF 628  TSF 192  CATAS 73397  CATAS 72059  CATAS 879  CATAS 78426  CATAS 87662  Mean value  Variation coefficient/% | 33.58 CDcd  38.53 Bb  31.46 CDd  33.90 CDcd  36.13 CBcb  33.10 CDcd  33.27 CDcd  31.20 Dd  34.26 CBDcd  45.92 Aa  35.13 + 4.35 Xx  12.37 | 27.74 Dd  35.45 BAba  29.20 DCd  28.87 DCd  27.58 Dd  30.93 BDCdc  34.48 BACbac  31.25 BDACbdc  31.59 BDACbdc  37.13 Aa  31.42 + 3.30 YXyxz  10.52 | 32.28 BACbc  38.70 Aa  28.17 Cc  33.63 BACbac  28.96 BCc  33.10 BACbac  32.33 BACbc  29.81 BCc  29.03 BCc  36.58 BAba  32.26 + 3.44 YXyx  10.68 | 29.97 BCbcd  37.38 Aa  26.00 Cd  32.66 BACbc  28.33 BCbcd  28.82 BCbcd  28.62 BCbcd  29.72 BCbcd  27.94 BCcd  33.03 BAba  30.25 + 3.27 YXZyz  10.82 | 30.52 BDCcd  36.41 BAb  28.00 Dd  33.12 BDCcb  27.70 Dd  28.58 Dcd  29.75 DCcd  29.62 DCcd  35.53 BACb  40.96 Aa  32.02 + 4.38 YXyxz  13.67 | 26.93 BCbc  33.63 Aa  24.40 Cc  27.93 BACbc  28.73 BACbc  29.73 BACba  25.03 BCc  24.43 Cc  31.05 BAba  30.23 BACba  28.21 + 3.07 YZdz  10.88 | 26.53 BDCc  33.45 BAa  19.02 Ed  25.28 DECc  27.65 BDACbc  24.40 DEc  18.92 Ed  19.30 Ed  32.19 BACba  34.28 Aa  26.10 + 5.89 Zd  22.56 | 29.65 Ccb  36.22 BAa  26.61 Cc  30.77 BCcb  29.30 Ccb  29.81 Ccb  28.91 Ccb  27.90 Ccb  31.66 BACb  36.87 Aa  30.77 + 3.35  10.89 |

difference at 0.01 and 0.05 levels, respectively; values followed by different uppercase letters ‘XYZ’ and lowercase letters ‘xyz’

within the same row denote significant difference at 0.01 and 0.05 levels, respectively.
