# Supplementary material for: The relationship between latex metabolism gene expression with rubber yield and related traits in Hevea brasiliensis
Source: BMC Genomics. 2018 Dec 10;19:897. doi: 10.1186/s12864-018-5242-4 (PMC6288877; doi:10.1186/s12864-018-5242-4)
Supplement: Supplementary file 4 — Table S4. Variability in dry rubber yield per tree per tapping from different cultivars and different harvest months. (DOC 33 kb) [file 12864_2018_5242_MOESM4_ESM.doc]

**Supporting Information**

**Table S4:** Variability in dry rubber yield per tree per tapping from different cultivars and different harvest months.

| Cultivars | Monthly mean dry rubber yield per tree per tapping (g) | | | | | | | |
| --- | --- | --- | --- | --- | --- | --- | --- | --- |
|  | May | June | July | August | September | October | November | Average |
| RRIM 600  PR 107  TSF 523  TSF 628  TSF 192  CATAS 73397  CATAS 72059  CATAS 879  CATAS 78426  CATAS 87662  Mean value  Variation coefficient/% | 29.39 EDCde  30.89 EDCde  16.42 EDFfe  48.12 BAba  23.66 EFfe  8.57 Fg  12.72 EFfg  24.62 BDCdc  41.49 BACbc  51.86 Aa  28.77 + 14.66 YZzy  50.96 | 32.57 BCbc  20.60 DCdc  14.29 DCdc  50.65 BAa  22.19 DCdc  3.89 Dd  6.98 DCd  19.90 BCbc  54.62 Aa  44.00 BAba  26.97 + 17.81 Zz  66.02 | 26.26 DCcb  24.07 DCcb  14.26 DCcb  49.77 Aa  18.12 DCcbd  7.96 DCcd  6.59 Dd  18.68 BCb  45.76 BAa  55.03 Aa  26.65 + 17.49 Zz  65.63 | 32.93 BACedc  31.53 BACedc  22.72 BACedc  46.39 BAbac  26.47 BCed  11.98 Ce  18.75 BCed  34.78 Aba  35.59 BACbdc  52.42 Aa  31.36 + 12.18 YXZxzy  38.84 | 46.68 CBDcb  35.36 CEBDced  35.50 CBcb  54.02 Bb  28.28 EDfe  11.49 Ef  15.06 CEDfed  31.50 CBDcbd  47.24 CBDcb  77.97 Aa  38.31 + 19.42 Xw  50.68 | 40.20 BDACbdc  25.82 Dd  23.48 Dd  55.97 BAba  36.94 BDCdc  29.91 DCd  35.71 BDCdc  61.37 Aa  41.12 BDACbdc  52.31 BACbac  40.28 + 12.77 YXxw  31.69 | 39.18 CBcb  37.68 CBDcb  19.19 De  40.60 CBcb  31.89 CBDcebd  26.07 CDced  22.24 CDed  33.93 CBDcbd  46.25 Bb  92.07 Aa  38.91 + 20.52 YXZxwy  52.74 | 35.31 BCDcd  29.42 EDed  20.84 EDegf  49.36 BAb  26.79 EDedf  14.27 Eg  16.86 Egf  32.11 CDed  44.58 BCcb  60.81 Aa  33.04 + 14.88  45.03 |

Values followed by different uppercase letters ‘ABCDEF’ and lowercase letters ‘abcdefg’ within the same column indicate significant difference

at 0.01 and 0.05 levels, respectively; values followed by different uppercase letters ‘XYZ’ and lowercase letters ‘wxyz’ within the same row

denote significant difference at 0.01 and 0.05 levels, respectively.
