# Supplementary material for: The relationship between latex metabolism gene expression with rubber yield and related traits in Hevea brasiliensis
Source: BMC Genomics. 2018 Dec 10;19:897. doi: 10.1186/s12864-018-5242-4 (PMC6288877; doi:10.1186/s12864-018-5242-4)
Supplement: Supplementary file 5 — Table S5. Diversity in monthly and annual dry rubber yields of individual plant of different cultivars. (DOC 35 kb) [file 12864_2018_5242_MOESM5_ESM.doc]

**Supporting Information**

**Supplementary Table S5.** Diversity in monthly and annual dry rubber yields of individual plant of different cultivars.

| Cultivars | Mean dry rubber yield per tree per month (g) | | | | | | | | kg·t^-1^·a^-1^ |
| --- | --- | --- | --- | --- | --- | --- | --- | --- | --- |
|  | May | June | July | August | September | October | November | Monthly average |  |
| RRIM 600  PR 107  TSF 523  TSF 628  TSF 192  CATAS 73397  CATAS 72059  CATAS 879  CATAS 78426  CATAS 87662  Mean value  Variation coefficient/% | 264.52 EDCde  278.04 EDCde  147.75 EDFfe  433.07 BAba  212.92 EFfe  77.16 Fg  114.50 EFfg  221.60 BDCdc  373.43 BACbc  466.70 Aa  258.97 + 131.98 YXZyz  50.96 | 260.52 BCb  164.77 DCcbd  100.05 DCcbd  405.22 BAa  177.56 DCcb  27.25 Dd  48.84 Dcd  139.29 DCcb  491.60 Aa  383.44 BAa  219.85 + 159.39 Zz  72.50 | 210.07 CBcb  192.58 CBcb  114.12 CBcb  398.13 Aa  144.96 CBcbd  63.70 CBcd  52.73 Cd  149.43 Bb  411.84 Aa  495.28 Aa  223.28 + 156.09 Zz  69.91 | 263.46 BAbdac  252.26 BAbdac  159.06 BAdec  371.14 Aa  211.74 BAde  83.87 Be  131.23 Bde  243.44 Abac  249.10 BAbdc  367.52 Aba  233.28 + 92.71 YZz  39.74 | 420.15 BCcb  318.26 CDced  248.53 BCDcd  486.14 BAb  254.50 ECDed  80.43 Ef  105.43 EDfe  220.50 CDced  377.92 BCDcbd  631.94 Aa  314.38 + 170.20 Xx  54.14 | 321.56 BCbdc  206.57 Cd  211.33 Cd  447.75 BAba  295.52 BCdc  269.20 BCd  321.36 BCbdc  552.30 Aa  328.97 BCbdc  466.95 BAbac  342.15 + 113.00 YXyx  33.02 | 313.42 CBcb  301.41 CBDcb  134.34 Ee  324.76 CBcb  255.09 CEBDcd  182.50 CEDde  155.66 EDde  237.53 CEBDcde  370.03 Bb  742.45 Aa  301.72 + 173.02 YXZyz  57.35 | 293.39 BCDcd  244.84 EFCDed  159.31 EFDegf  409.46 BAb  221.76 EFDedf  112.02 Fg  132.82 EFgf  252.01 ECDed  371.84 BCcb  507.75 Aa  270.52 + 127.46  47.12 | 2.05 DCc  1.71 DEdc  1.12 DEFde  2.87 BAb  1.55 DEFdce  0.78 Ff  0.93 EFfe  1.76 DCc  2.60 BCb  3.55 Aa  1.89 + 0.89  47.12 |

kg·t^-1^·a^-1^: dry rubber yield by kilogram per tree per year.

Values followed by different uppercase letters ‘ABCDEF’ and lowercase letters ‘abcdefg’ within the same column indicate significant difference at 0.01 and 0.05 levels, respectively; values followed by different uppercase letters ‘XYZ’ and lowercase letters ‘xyz’ within the same row denote significant difference at 0.01 and 0.05 levels, respectively.
